# Rapid analysis of meat floss origin using a supervised machine learning-based electronic nose towards food authentication

**DOI:** 10.1038/s41538-023-00205-2

**Published:** 2023-06-16

**Authors:** Linda Ardita Putri, Iman Rahman, Mayumi Puspita, Shidiq Nur Hidayat, Agus Budi Dharmawan, Aditya Rianjanu, Sunu Wibirama, Roto Roto, Kuwat Triyana, Hutomo Suryo Wasisto

**Affiliations:** 1 PT Nanosense Instrument Indonesia, Yogyakarta, 55167 Indonesia; 2 Department of Physics, Faculty of Mathematics and Natural Sciences, Universitas Gadjah Mada, Sekip Utara PO Box BLS 21, Yogyakarta, 55281 Indonesia; 3 Indonesian Oil Palm Research Institute, Jalan Taman Kencana No 1, Bogor, 16128 Indonesia; 4 Faculty of Information Technology, Universitas Tarumanagara, Jl. Letjen S. Parman No. 1, Jakarta, 11440 Indonesia; 5 Department of Materials Engineering, Institut Teknologi Sumatera, Terusan Ryacudu, Way Hui, Jati Agung, Lampung, 35365 Indonesia; 6 Department of Electrical and Information Engineering, Universitas Gadjah Mada, Jl. Grafika 2, Yogyakarta, 55281 Indonesia; 7 Department of Chemistry, Faculty of Mathematics and Natural Sciences, Universitas Gadjah Mada, Sekip Utara PO Box BLS 21, Yogyakarta, 55281 Indonesia; 8 Institute of Halal Industry and System (IHIS), Universitas Gadjah Mada, Sekip Utara, Yogyakarta, 55281 Indonesia

**Keywords:** Chemical engineering, Characterization and analytical techniques, Mass spectrometry, Biological physics

## Abstract

Authentication of meat floss origin has been highly critical for its consumers due to existing potential risks of having allergic diseases or religion perspective related to pork-containing foods. Herein, we developed and assessed a compact portable electronic nose (e-nose) comprising gas sensor array and supervised machine learning with a window time slicing method to sniff and to classify different meat floss products. We evaluated four different supervised learning methods for data classification (i.e., linear discriminant analysis (LDA), quadratic discriminant analysis (QDA), k-nearest neighbors (k-NN), and random forest (RF)). Among them, an LDA model equipped with five-window-extracted feature yielded the highest accuracy values of >99% for both validation and testing data in discriminating beef, chicken, and pork flosses. The obtained e-nose results were correlated and confirmed with the spectral data from Fourier-transform infrared (FTIR) spectroscopy and gas chromatography–mass spectrometry (GC-MS) measurements. We found that beef and chicken had similar compound groups (i.e., hydrocarbons and alcohol). Meanwhile, aldehyde compounds (e.g., dodecanal and 9-octadecanal) were found to be dominant in pork products. Based on its performance evaluation, the developed e-nose system shows promising results in food authenticity testing, which paves the way for ubiquitously detecting deception and food fraud attempts.

## Introduction

Nowadays, various kinds of meat products are widely manufactured and consumed throughout the world^1^. The consumption of meat, especially pork, turns out to be highly critical when it comes to the potential risks of having allergic diseases^2,3^. Pork-containing ingredients have transmitted major meat-based parasites including protozoa (e.g., Toxoplasma gondii and Sarcocystis spp.) and helminths (e.g., Trichinella spp. and Taenia spp.) that cause serious illness and death^4^. On the other hand, religious belief on halal foods has become an important reason for strict restriction of pork-containing ingredients in various foods^5,6^. Therefore, it is important to develop an accurate system to authenticate the origin of food products and to ensure consumer protection on daily consumption of meat-based foods.

Several methods for identifying pork in the meat products have been explored and implemented by different research groups and food communities, which mainly used commercial tools based on biochemical detection techniques (e.g., differential scanning calorimetry (DSC)^7^, enzyme-linked immunosorbent assay (ELISA)^8^, nuclear magnetic resonance (NMR)^9^, and polymerase chain reaction (PCR)^10,11^). However, these methods can only be tested in a well-equipped laboratory, in which many technical limitations are associated with sample preparation, calibration of the instrument, personnel and training costs, and laboratory infrastructure requirements^12,13^. Meanwhile, it is necessary to use a mobile and easy-to-use instrument that can rapidly detect the pork content in the meat products (e.g., meat flosses) without reagents and complex preparation.

The volatility of meat products depends on their volatile organic compound (VOC) compositions, as they emit specific odors^14^. The VOCs can be divided into several groups, i.e., organic acids, alcohols, esters, aldehydes, hydrocarbons, terpenes, furans, and others^5^. The presence of the VOCs determines the aroma of a particular product^15^. Sources of volatile compounds can also be found in spices and other food additives, which contribute to the overall taste and modulate certain authoritative reactions^5^. Therefore, VOC information can be further processed for identification of meat floss origin.

An electronic nose (e-nose) is an aroma analyzer comprising gas sensor array and artificial intelligence to emulate human olfactory system. This system has recently been reported to be a versatile tool for different VOC-based sensing applications, e.g., monitoring of toxic air pollutants^16^, fast screening of coronavirus disease 2019 (COVID-19)^17,18^, and assessment of food authenticity and adulteration^19–24^. In addition, e-nose can sense the odor patterns from substances present in the food products including chicken^25–27^, pork^28^, goat meat^26^, fish^29^, duck, and goose meat^27,30^. E-nose offers several advantages compared to other nondestructive technologies that have been explored in meat quality and safety evaluation (e.g., computer vision, spectroscopy, hyperspectral imaging, and multispectral imaging^30^). For instance, e-nose provides high sensitivity and accuracy, produces rapid result classification^27^, requires simple sample preparation, and has economical procedure of usage^30^. The system also consumes low power^31^, provides high portability^32^, does not require skilled laboratory operators^26^, and implements pattern-based detection^33^.

A further enhancement of pattern recognition models is necessary in e-nose for data analysis besides fabrication and optimization of highly sensitive low-power gas sensors^34–40^. To obtain high accuracy and precision, e-nose requires appropriate learning algorithms to extract and to learn complex patterns detected by its sensor components. This is critical for the system to classify or to discriminate between different meat flosses. Principal component analysis (PCA) is a well-known technique used not only for dimensionality reduction, but also for feature extraction and identification^41^. This unsupervised technique transforms the signals into a lower dimensional matrix to calculate the Eigenvector and Eigenvalue and to find the greatest Eigenvalues as the unique feature^42^. To perform the identification, various supervised classification models can be used in classifying the extracted features, in which they have their certain unique advantages^29,43–46^. For instance, the linear discriminant analysis (LDA) possesses an advantage of low computational cost due to the reduction of dimensional complexity of the features. Meanwhile, the quadratic discriminant analysis (QDA) is relatively more flexible but has a slightly higher computational cost than LDA. Furthermore, while the k-nearest neighbors (k-NN) algorithm is quite robust to noisy training data due to its instance-based learning, random forest (RF) model offers a benefit of less frequent overfitting because it comprises several decision trees. In addition, all those four methods (LDA, QDA, k-NN, and RF) have simple architectures and do not require any training or long iteration processes to perform optimal classification. Hence, the complexity of e-nose system can be reduced. Although these models can be classified as simple and old machine learning models, in several cases they still outperform other newer and more complex machine learning models.

In this work, we developed a compact portable e-nose comprising gas sensor array and integrated machine learning models to identify and to classify the origins of different meat floss products. To enhance the system accuracy, a window time slicing method was applied during the feature extraction process, in which the optimum number of windows was also investigated. The obtained data were analyzed not only using the unsupervised learning model of PCA, but also utilizing four supervised learning models (LDA, QDA, k-NN, and RF). The performances of machine learning models were evaluated for discriminating beef, chicken, and pork meat flosses. In addition to learning optimization and data analysis, the investigation of meat floss using e-nose was also supported with two spectral material characterizations (i.e., Fourier-transform infrared (FTIR) spectroscopy and gas chromatography–mass spectrometry (GC-MS)), which were valuable to validate the e-nose and elucidate the possible detected VOCs in this study.

## Results and discussion

### Portable electronic nose system

The developed e-nose comprises eight metal-oxide semiconductor (MOS) gas sensors enabling detection of various target gases, as listed in Table 1. Despite their high robustness, all sensors are sensitive to more than a single detectable gas, which is a typical characteristic for such inorganic MOS materials (i.e., low selectivity)^47–49^. In another study, similar types of commercial MOS sensors were used for real-time classification of black tea according to its quality level^50^. A chemoresistive gas sensor employing thin-film MOS relies on changes in the conductivity of the material caused by the adsorption of target gas molecules on its surface^51^. More specifically, it works based on the depletion layer variation at the grain boundaries in the presence of oxidizing or reducing gases, which result in modulation in the height of the energy barrier for free charge carriers to flow. Hence, the resistance of the sensing material will change. Here, the gas molecules that interact with the MOS material act as either donor or acceptor for the charge carriers altering the MOS resistivity.

**Table 1:** Specification of all chemoresistive sensors used in the e-nose system.

Gas sensor	Sensor type	Main target analytes	Measurement range	Sensitivity (sensor resistance ratio change)	Limit of detection (LOD) (ppm)	Detectable gases (cross-sensitivity)
S1	MOS	Organic solvent vapors	1 to 10 kΩ in ethanol at 300 ppm/air	0.40 ± 0.10	50	Acetone, benzene, carbon monoxide, ethanol, isobutane, methane, and *n*-hexane
S2	MOS	Isobutane methane, and propane	1 to 25% lower explosive limits (LEL) of each gas	0.50 to 0.65	300	Butane, methane, and propane
S3	MOS	Alcohol and solvent vapors	50 to 5000 ppm ethanol	0.3 to 0.5 in ethanol	50	Alcohol and carbon monoxide
S4	MOS	Chlorofluorocarbons	4 to 40 kΩ in 1,1,1,1-tetrafluoroethane (R-134a) at 100 ppm/air	0.50 to 0.65	10	Chlorofluorocarbon, ethanol, and hydrofluorocarbons
S5	MOS	Ammonia	30 to 300 ppm	0.55 ± 0.15	30	Ammonia, ethanol, hydrogen, and isobutane
S6	MOS	Air contaminants (trimethylamine and methyl mercaptan)	1 to 100 ppm ethanol	<0.5	0.3	Ethanol, hydrogen, hydrogen sulfide, methyl mercaptan, and trimethylamine
S7	MOS	Air contaminants (hydrogen and ethanol)	1 to 30 ppm of H_2_	0.3 to 0.6	1	Carbon monoxide, ethanol, hydrogen, isobutane, and methane
S8	MOS	Combustible gases	5 to 15 kΩ in methane at 1000 ppm/air	0.6 ± 0.05	500	Carbon monoxide, ethanol, hydrogen, isobutane, methane, and propane

Sensors are based on metal-oxide semiconductor (MOS) materials and obtained from a commercial sensor manufacturer. In terms of selectivity, each sensor sensitively reacts to more than a single target gas. The detectable gases indicate the cross-sensitivity of sensors toward several different gases.

**Fig. 1:**
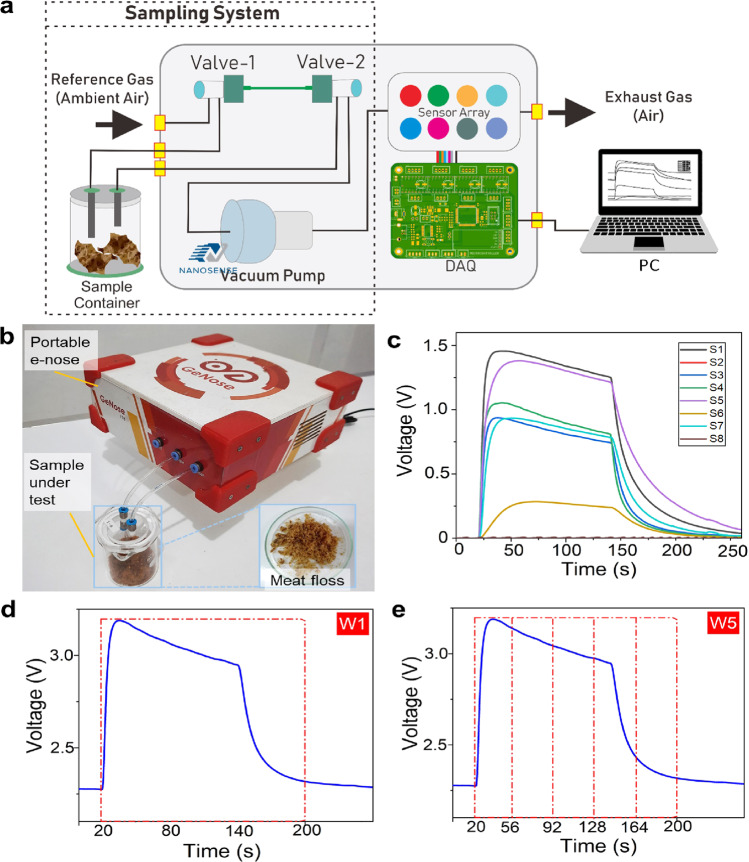
Portable electronic nose (e-nose) for meat floss authentication. **a** Configuration of the e-nose comprising a sampling system, a sensor array, and a data acquisition (DAQ) system. **b** Photograph of e-nose during test of meat floss. The meat floss sample was placed inside a sampling chamber during aroma test. The headspace system has two processes (i.e., sensing and purging), in which through data processing and pattern recognition, the aroma of meat floss samples can be identified. **c** Typical signals obtained from eight sensors (S1–S8) in the e-nose. For each measurement, three phases are defined: a time delay phase of 20 s, a sampling phase of 120 s, and a purging phase of 120 s. Time window slicing method is implemented in the sensing signal: **d** one window (W1) and **e** five windows (W5).

At the system level, the change in resistance is converted into voltage using a voltage divider circuit. The measured analog voltage from each sensor is then converted to digital form by 16-bit analog-to-digital converter (ADC). In the data acquisition system (DAQ), the microcontroller reads digital data for every 100 ms. The data are then sent to the computer via the RS232 serial protocol for analysis. The complete experimental setup of e-nose for meat floss authentication is depicted in Fig. 1a, b, and Supplementary Fig. 1.

The e-nose system involves two chambers, i.e., a sampling chamber to insert the tested samples and a sensing chamber to mount the sensors detecting different VOCs. Eight sensors (S1–S8) were employed inside the sensing chamber to interact with VOC molecules released by the meat floss having a certain concentration level. The total assessment time was set at 260 s for each meat floss sample investigation consisting of delay, sampling, and purging phases of 20 s, 120 s, and 120 s, respectively. During the sampling phase, reference air was flown from the reference connector to the first valve passing through the sampling chamber containing two grams of meat floss sample and subsequently entering the sensing chamber via the second valve. During the delay and purging phases, reference air was supplied from the reference connector to the first valve and passed directly to the sensing chamber via the second valve. The difference between these two phases is the gas type that is being sensed. At the delay phase, ambient air was sensed. Meanwhile, at the purging phase, residual sample gas was detected and drawn out from the sensing chamber. The typical signals from all eight sensors (S1–S8) during an assessment of meat floss are depicted in Fig. 1c. It is obvious that during the sampling phase, the sensors yield different sensing signal characteristics, which depend on their sensitivity and selectivity towards target gases (see Table 1).

The selection criteria of the employed sensors were based on their abilities to possibly detect the potential compound-based biomarkers in meat and their compatibility to be integrated with other controlling electronic components in the system. From several reports associated with the studies of mass spectrometry techniques for analyzing meat biomarkers^52–55^, main volatile compounds could be derived from beef (e.g., hydrocarbon [5-ethyl-m-xylene and 3-ethyl-2-methyl-1,3-hexadiene], and aldehyde [benzaldehyde]), chicken (e.g., hydrocarbon [b-cymene], alcohol [2-pentanol], and aldehyde [3-methyl-butanal]), and pork (e.g., aldehyde [pentanal], hydrocarbon [2,6-dimethylcyclohex anone, 2,4,5-trimethyl-thiazole, 5-ethyl-3-(3-methyl-5-phenylpyrazol-1-yl, 1,2,4-triazol-4-amine)], and alcohol [1-undecanol, cyclobutanol])^54^.

The main VOC species in dried pork slices include alcohols, aldehydes, acids, ketones, heterocyclic compounds, aromatic hydrocarbons, and esters^53^. Beef contains several metabolites (decanoic acid, uric acid, elaidic acid, and 3-phosphoglyceric acid)^52^. Meanwhile, the GC-MS results of this study showed that the beef, chicken, and pork floss samples contained hydrocarbons (2,4-dimethylhept-1-ene, 4-methyl-1-decene, (E)-4-dodecene, (Z)-5-tridecene, and 3-trifluoroacetoxytridecane), which were also able to be detected by six sensors in e-nose (i.e., S1, S2, S4, S5, S7, and S8). Besides hydrocarbons, e-nose can detect alcohol compounds (2-butyl-1-octanol, 2,4-di-tert-butylphenol, ethyl iso-allocholate, 6,11-dimethyl-2,6,10-dodecatrien-1-ol) by S3. The content of ether (6-methylheptyl vinyl ether) was able to be measured by S1, S3, S7, and S8. To distinguish chicken from other samples through the hydrocarbon compound (6-methyl-octadecane), S1, S2, S4, S5, S7, and S8 could be used. The capability of S1, S3, S7, and S8 to sniff ether compounds also plays a role in finding the methoxyacetic 2-tridecyl ester compound, which is the characteristic of chicken. The hydrocarbons (1-methylhexyl hydroperoxide and 4-ethyl-octane), which were detected only in beef and chicken, were not found in the pork. The characteristics of pork that could be sensed by S3 were alcohol compounds (i.e., trans-2-dodecen-1-ol and 12-methyl-E,E-2,13-octadecadien-1-ol). Meanwhile, aldehydes (octadecanal and 9-octadecanal) were detected by S1, S2, S5, S7, and S8.

Moreover, different types of meat flosses resulted in altered sensor signals. To provide comparable trend among different measurements, the raw signals were then normalized according to their initial baselines (see Supplementary Fig. 2). In this experiment, the baseline normalization process was used to normalize the signals from different measurements for an effective comparison^56^.

The baseline-corrected statistical analysis was processed by subtracting all the original sensor responses with the first response recorded in the delay phase^57^, which can be represented by the following equation:

1 $${V^{\prime} }_{{ij}}={V}_{{ij}}-{V}_{i0}$$

where ${V^{\prime} }_{{ij}}$ is the normalized response or signal of the sensor *i* in timestamp *j* and ${V}_{i0}$ is the sensor *i* response at timestamp of 0 s.

To further improve the e-nose system performance, the time window slicing method was implemented to create the separating detection lines in the disturbances signal. For a single window (i.e., one window), data selection using this technique started from 20 to 200 s, in which the data were then processed to extract out features of maximum, minimum, mean, and median values (see Fig. 1d). For more windows (e.g., 2–6 windows), a further time slicing was implemented in the single defined window of the data (see Fig. 1e and Supplementary Fig. 3).

### Unsupervised clustering by principal component analysis (PCA)

In contrast to signals produced by conventional analytical instruments (e.g., GC-MS), e-nose signals generally do not correspond directly to specific chemical components. Instead, the pattern recognition method is required to extract and to analyze information from the sensing signals generated by the sensor arrays. Principal component analysis (PCA) is an unsupervised, non-parametric statistical technique, which is commonly used as the first step for exploring spectral data^58,59^. This technique linearly reduces the large number of variables in the original data into principal components, which contain most of the variable data.

**Fig. 2:**
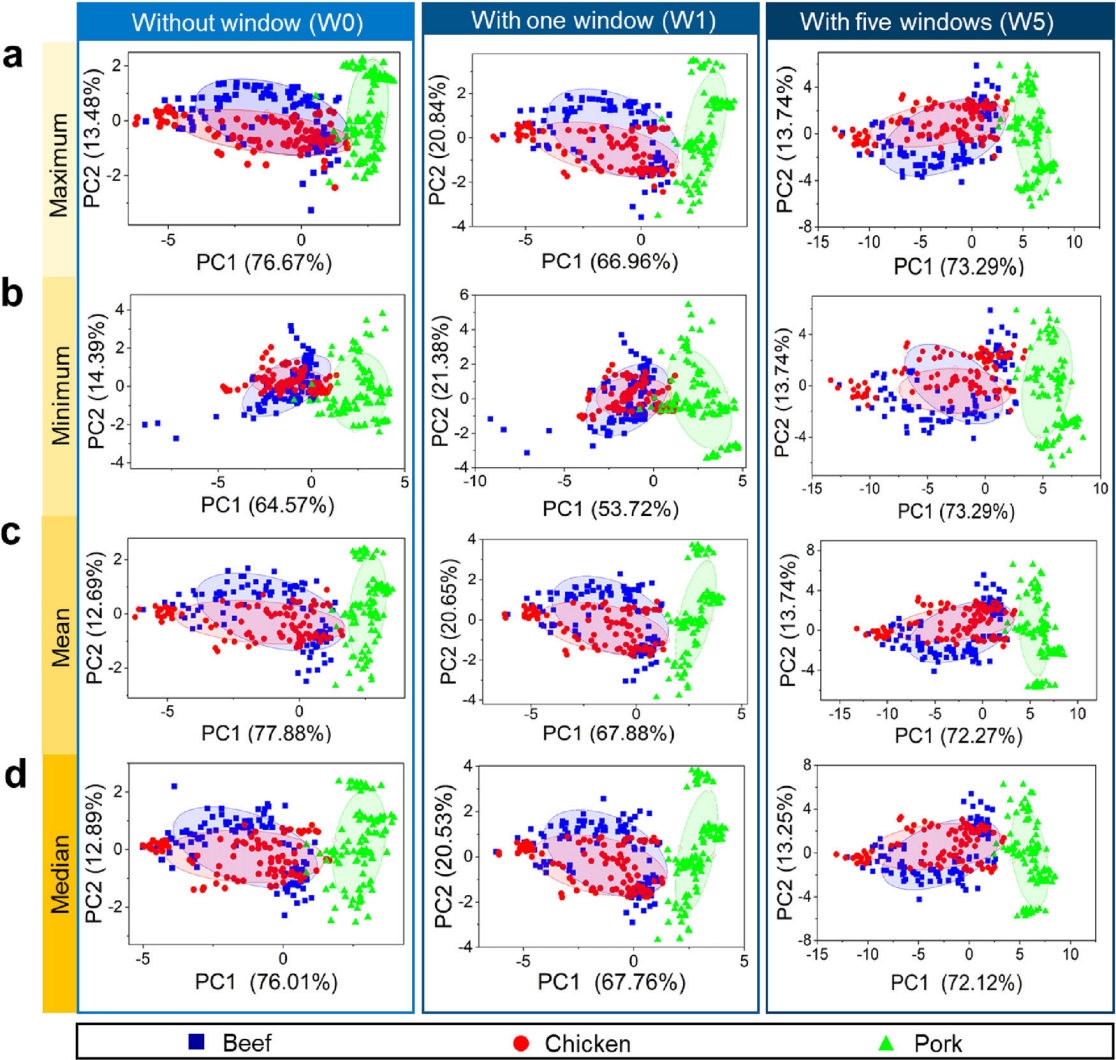
Unsupervised classification of meat flosses using principal component analysis (PCA) integrated with time window slicing method. PCA is implemented to analyze output sensing signals that are preprocessed with four different extracted features: **a** maximum, **b** minimum, **c** mean, and **d** median values. Time window slicing method is applied to construct different window numbers in the data (i.e., 1 window (W1) and 5 windows (W5)). The condition without window (W0) is also analyzed as reference. PCA can create separated clusters between pork and non-pork meat flosses in the data measured by e-nose, despite the existing overlap between beef and chicken classes.

In the first step of data analysis, we implemented PCA to perform clustering of three different labels, which are beef floss, chicken floss, and pork floss. Each sample was made of single meat origin. Thus, beef, chicken, and pork were not mixed into one composition during meat floss production. The PCA visualization is shown in Fig. 2, where we varied the feature extraction used for the analysis. We employed the maximum, minimum, mean, and median of the e-nose sensor responses. The maximum value indicates the highest sensor reaction rate in response to the sample aroma. The minimum value describes the lowest response value from the entire data. The mean value is the ratio between the total sensor response and the total data. The median value indicates the middle value of the overall sensor response^60^. The four extracted features were obtained using the formulas listed in Table 5. Another variation implemented in the analysis was the amount of sliced time windows. The number of features was also related to the number of windows, in which each window provides maximum, minimum, mean, and median values. Here, the time window slicing method was implemented up to 6 windows to find the optimum window number for the resulting dataset. The methods having no window (W0), one window (W1), and five windows (W5) yielded 8, 10, and 42 proportions of variance, respectively. The detailed results for other window numbers are shown in Supplementary Fig. 4 and Supplementary Fig. 5. Normally, the PCA is interpreted by visualizing the principal component (PC) scores. The scores of the first two PCs from sets 1 and 2 are referred to as PC1 and PC2, respectively, which represent the largest proportion of data variance.

Figure 2a–d represents the score plots of meat flosses (beef, chicken, and pork) using extracted features of maximum, minimum, mean, and median values with the first two PCs (PC1 and PC2) accounting for 87%, 87%, 86%, and 85% of the total variance, respectively. The plots show that the pork samples differ spectrally, as the PCA can distinguish the samples in separated clusters. The samples are grouped into three different classes, indicating that the components of the beef and chicken classes are very similar and separated from the pork class.

From the PCA results, the beef and chicken clusters are plotted in the negative quadrant, while the pork cluster is in the positive quadrant of the PC1. Here, the pork is diverged to form a wide angle from the other two materials. This indicates that the pork has a volatile content, which is directly distinguishable from other meat types. On the other hand, the beef and chicken clusters are still overlapped in the PC1, despite the increase in the number of windows. The overlap between beef and chicken is explained by the GC-MS results in Table 4 where both contain numerous similar volatile compounds (e.g., 1-methylhexyl hydroperoxide and 4-ethyl-octane). Although beef and chicken classes still possess overlap in the PC1, they exhibit unique volatile component characteristics that can be distinguished in different quadrants of the PC2. Here, the beef is separated in the negative quadrant, while the chicken is in the positive quadrant. This is due to the unique volatile compound characteristics in the chicken (e.g., 6-methyl-octadecane and methoxyacetic 2-tridecyl ester).

Again, although PCA can demonstrate the initial potential for cluster formation among the different meat flosses, the overlapping results between beef and chicken clusters cause difficulties in better classification and identification. Therefore, the supervised learning method was required to be used for finding out the specific variation and classifying the three classes of meat flosses.

### Multivariate supervised learning for enhanced classification

Highly accurate detection of certain chemical compounds from the multivariate sensor readout can be interpreted robustly using powerful supervised learning methods. In our study, four different supervised learning methods were used, i.e., linear discriminant analysis (LDA), quadratic discriminant analysis (QDA), k-nearest neighbors (k-NN), and random forest (RF).

LDA was applied to reduce dimensionality and to identify linear combination of features to distinguish two or more classes of meat floss odors. In case there are three classes of odors, LDA creates one hyperplane and projects the data in such a way that the separation of classes is maximized. The hyperplane is drawn by minimizing the distance within the same class and maximizing the distance between the classes^61^. Cross validation was performed for the LDA model to evaluate its robustness in predicting the meat floss. Before statistical analysis, matrix data were subjected to standardization procedures (scaling and centering). After performing standardization, the initial database was divided into two groups (i.e., a training data subset and a testing data subset). The database used in this research comprises 300 data (i.e., 100 data for each type of meat floss). The database was then divided into a 75% training dataset and a 25% testing dataset, which was carried out by random sampling method. The training data were used to develop a classification model. Here, the chosen model was validated using 10-fold cross validation. The testing data subset was utilized for external validation—estimating classification accuracy using unseen data.

In this experiment, no standard rule was applied for splitting between training and testing data. There is no clear evidence that suggests machine learning models always deliver stable performance under specific data splitting configuration. Thus, the data splitting cannot be decided a priori^62^. Importantly, the ratio between training and testing data should be set enabling the model to predict the unseen data in this study. We split the training and testing data into 75% and 25% ratio, respectively. We opted to use this ratio to ensure that the models were able to gain sufficient information from the training data before being evaluated with unseen data in the testing set. Note that the testing data were used in the external validation. For model selection, we separated the 75% training data into training and internal validation set with ratio of 75% and 25%, respectively.

Table 2 lists the accuracy values resulting from the validation and testing processes of various extracted statistical features (i.e., maximum, minimum, mean, and median values) using the LDA model. Validation accuracy is used as a reference to verify that the generated model does not exhibit overfitting. The testing accuracy is used to measure the ability of the generated model in predicting the unseen data. The LDA results indicate that the sensing signals captured by the e-nose can be used to discriminate the pork meat floss from its non-pork counterparts (beef and chicken meat flosses).

**Table 2:** Accuracy values resulting from validation and testing with the LDA model.

Window number	Accuracy (%)							
	Maximum		Minimum		Mean		Median	
	Validation	Testing	Validation	Testing	Validation	Testing	Validation	Testing
0	95.5	92.0	89.0	90.7	91.5	93.3	93.3	90.7
1	95.6	90.7	97.7	90.7	96.9	94.7	97.8	93.3
2	92.3	90.7	99.0	94.7	97.4	97.3	100	97.3
3	92.3	90.7	99.6	100	98.6	100	99.2	100
4	92.3	90.7	99.2	100	98.8	100	100	100
5	99.9	100	99.7	100	99.6	100	99.4	100
6	99.4	100	99.2	100	99.3	100	98.7	100

Validation accuracy was used during development of machine learning model under cross-validation. Testing accuracy was employed to evaluate the developed machine learning model on the unseen data. Different window numbers were evaluated using the LDA model (W1–W6).

Maximum, minimum, mean, and median statistical values were applied to extract the unique response shape of e-nose readout. In this approach, several features were extracted from the trapezoidal-shaped responses of e-nose. The responses were divided into three main types based on the planned further processing: original response curve, curve fitting parameters, and transform domain^63^. From Table 2, the highest average validation occurs in LDA with five windows (W5). The maximum, minimum, and mean scores reach accuracy values of 99.9%, 99.7%, and 99.6%, respectively. However, for the median, its highest accuracy value of 100% is found in the LDA with two (W2) and four windows (W4). For the testing results, the highest and the most stable accuracy values (100%) were obtained on maximum, minimum, mean, and median values with five (W5) and six windows (W6). Despite good results in testing, the accuracy values in the internal validation of LDA with W6 slightly dropped compared with those of LDA with W5. For instance, the accuracy of maximum metric decreased from 99.9% to 99.4% when six windows were implemented during feature extraction. These results indicate that the optimum number of windows should be investigated under different processed sensing signals and datasets. Our results suggest that increasing the number of windows does not always correspond to improvement of internal validation accuracy.

**Fig. 3:**
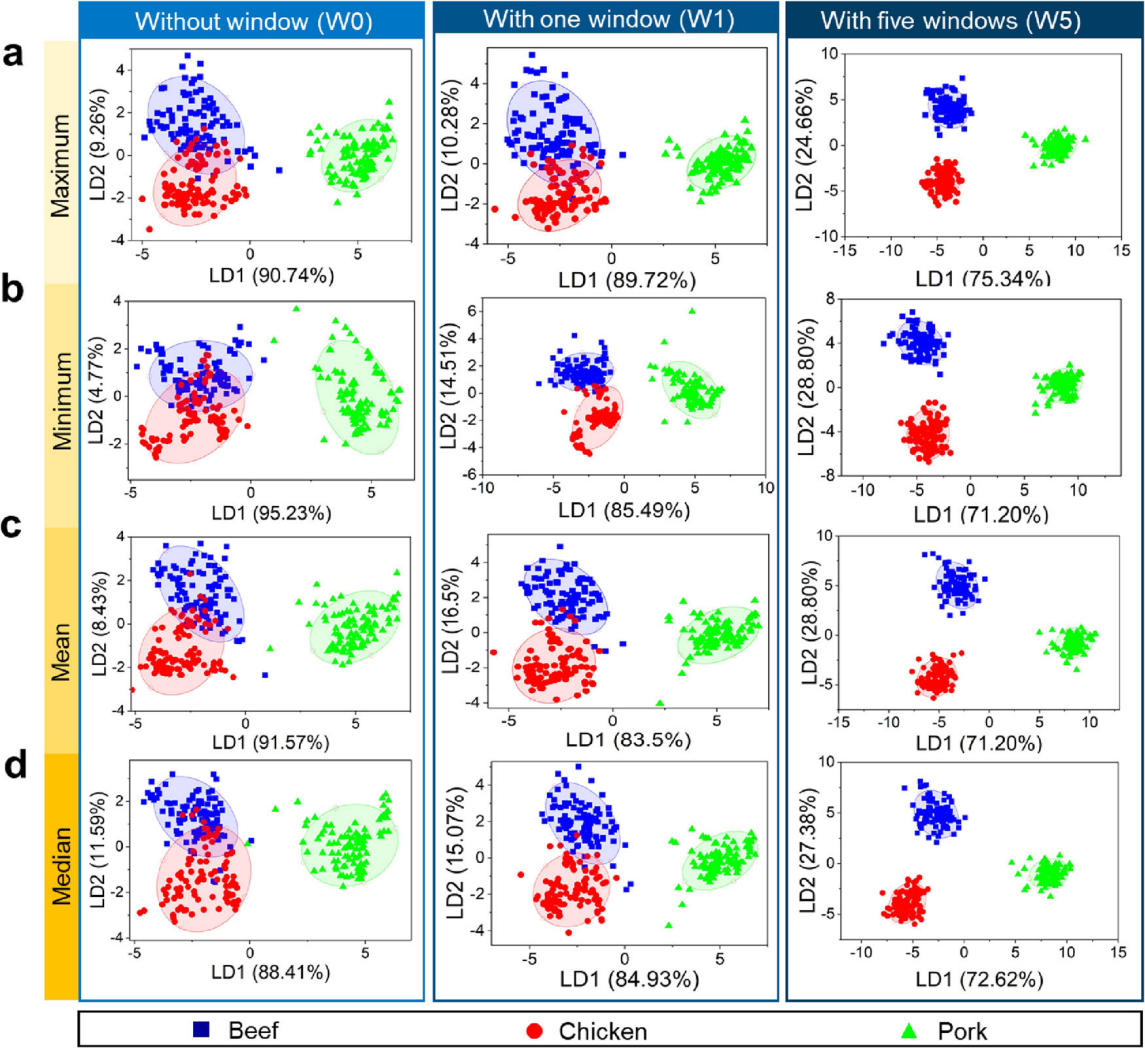
Supervised classification of meat flosses using linear discriminant analysis (LDA) integrated with time window slicing method. LDA was employed to analyze output sensing signals that were preprocessed with four different extracted features: **a** maximum, **b** minimum, **c** mean, and **d** median values. Time window slicing method was applied to construct different window numbers in the data (i.e., 1 window (W1) and 5 windows (W5)). The condition without window (W0) was also analyzed as reference. A clear cluster separation among three different meat flosses (beef, chicken, and pork) was yielded by the LDA at W5.

Figure 3a–d shows the classification of meat flosses using the LDA model integrated with different window numbers for all four extracted features (maximum, minimum, mean, and median values). Compared with other supervised models, the LDA reduced the complexity of the dimension. Hence, it is useful to investigate the distribution of each label in a lower dimension. In addition, LDA can be combined with other classification methods to provide additional advantage, where the extracted features from the LDA are considered labels in the training data. Hence, the data were more separated between different classes. This advantage yielded higher performance of the machine learning model^64,65^. Here, the classification is made with more than one canonical variable. The most significant variables are described by LD1 and LD2.

In Fig. 3, beef cluster tends to be distributed in the positive LD2 and negative LD1 quadrants, while the chicken cluster is distributed in the negative LD1 and LD2 quadrants. In contrast, the pork is clustered perfectly in the positive LD1 quadrant, while its LD2 spreads into positive and negative quadrants. Based on these results, three different types of meat flosses can be well separated with an external validation (testing) accuracy of >90%. Indeed, the lower dimensional features resulting from LDA simplify the analysis in machine learning algorithm by reducing insignificant and redundant features^66^. In comparison to the PCA approach, the LDA method is also capable of separating pork cluster from other material clusters at the LD1 stage. We implemented a time slicing window approach to reduce existing overlap at W0. Consequently, we obtained significant improvement in discrimination of beef and chicken clusters. The LDA-based classification results of the meat flosses using different window numbers (i.e., from W0 to W6) are displayed in Fig. 3, Supplementary Fig. 6, and Supplementary Fig. 7.

Although the plots from the feature extractions without window (W0) and with one window (W1) still indicate overlap between beef and chicken, the results still can be used to classify pork and non-pork meat flosses. W0 and W1 produce similar results because they process the data as one piece of information, in which the only difference is the duration of signal retrieval. The signals are divided into separated ranges by increasing the window number to extract different information. Based on the results, LDA can completely separate three clusters in the feature extraction using W5. Meanwhile, W6 can also separate the three samples, but its accuracy values are smaller than those of W5 (see Table 2).

The analysis process was performed with several window configurations to find the optimum number of windows. We aimed to achieve the highest internal and external validation accuracy values (see Supplementary Table 1). The time window slicing process affected the model on gaining insight into the data due to the natural response of the sensors. In e-nose, the gas sensing pattern usually has a trapezoidal shape with a specific area. The curve of the sensor response starts rising significantly when the adsorbed gas molecules have induced increase/decrease in the resistance of the MOS sensor (see Fig. 1c–e). Afterward, the curve will be stable at a certain voltage or resistance value for some time during sampling. Finally, the curve declines to the baseline state when the target gas molecules have been removed from the sensor active material surface. These unique and specific regions may offer more valuable insight if they are processed as individual parts. One approach that can be applied is the rising window method, which only focuses on the early part of the data for classification^67^. The advantage of using specific time window regions in data is that each sliced data region will be optimum because of its own unique characteristic^68^. In our case, when we started to apply four windows (W4) to our data during analysis using LDA model, we could identify the cluster separation between pork and non-pork clusters. However, the maximum value approach still suffered from overlapping beef and chicken clusters. After implementing five windows, all beef, chicken, and pork samples could be properly separated (see Fig. 3). This has proven that increasing the number of windows will enlarge the distance among the clusters. In this approach, the separation process was conducted by the Euclidean distance to calculate the interpoint distances between samples.

The Euclidean distance was reported efficiently for data clustering^69,70^. Euclidean distance has also been used as the main construction component for several other learning methods. For example, Euclidean distance was also used in the Ward’s method—a special type of agglomerative hierarchical clustering analysis (HCA)^71,72^. Euclidean distance is defined by the following equation:

2 $${d}_{{ik}}={\left[\mathop{\sum }\limits_{j=1}^{p}\left({V}_{{ij}}-{V}_{{kj}}\right)\right]}^{\frac{1}{2}}$$

where *d*_*ik*_ is the distance between clusters *i* and *k*, in which it is determined as the summation of square roots of each feature distance between clusters *i* and *j*. Meanwhile, *V* is the sensor response or extracted feature. To represent *p* feature or to make *p* dimension simpler, dimension reduction can be carried out using PCA, which is calculated as

3 $$P{C}_{i}=\mathop{\sum }\limits_{j=1}^{p}{a}_{{ij}}{V}_{j}$$

where *PC*_i_ is the principal component at a certain number *i*, which is obtained from the summation result of the multiplication of the multiplier coefficient *a* with the sensor response *V*.

Dimension reduction from *p* number of features combined with the Euclidean distance method is used to evaluate the effect of window variation on the distance separation between clusters. The distance between two clusters is calculated from the central point of each cluster, in which this has normally been termed as a pitch.

**Fig. 4:**
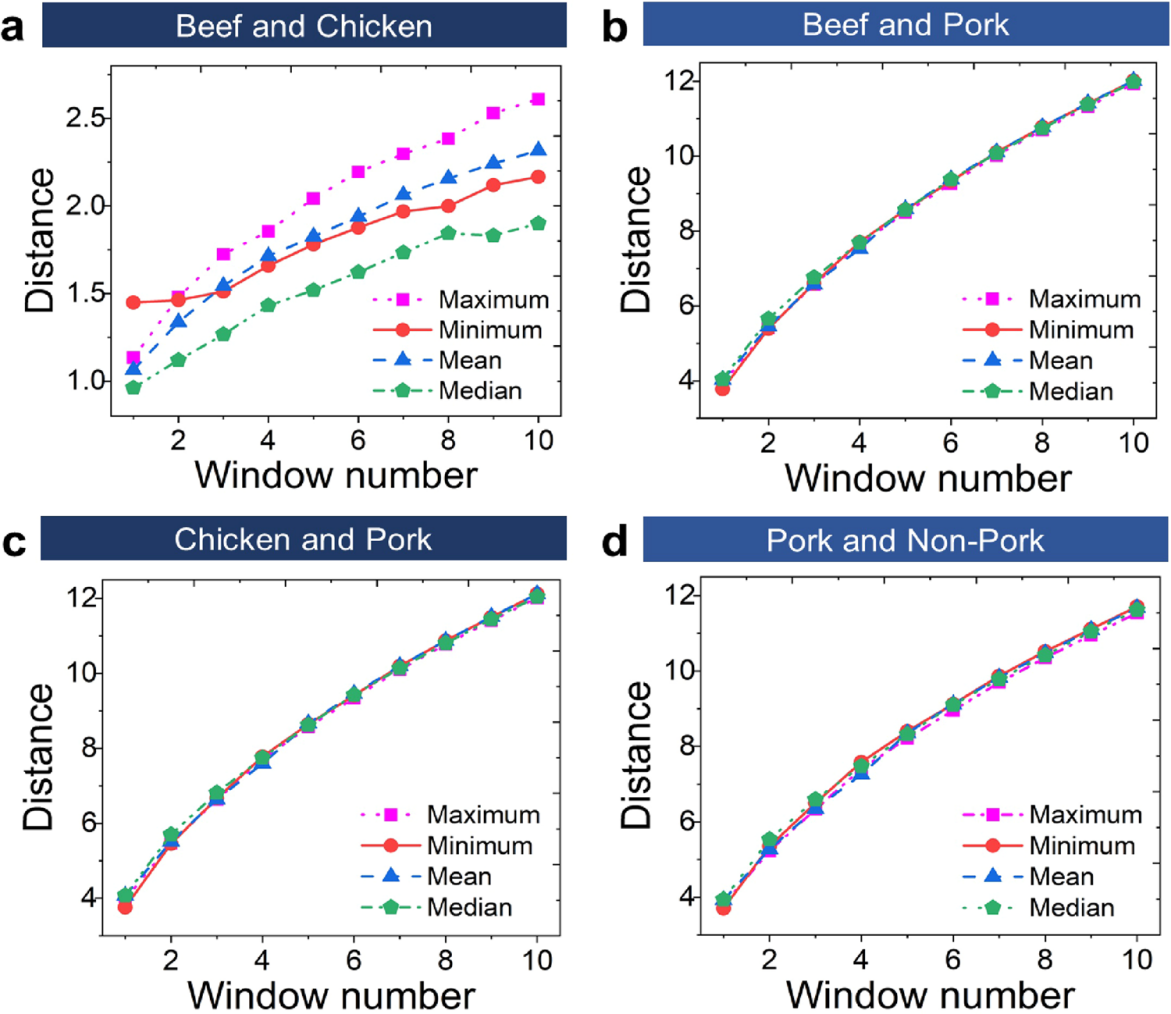
Effect of window number variation on distance between two separated different clusters. Evaluation of relationship between window number and cluster distance is applied to investigate the behaviors of **a** beef–chicken, **b** beef–pork, **c** chicken–pork, and **d** pork–non-pork meat floss clusters. Beef and chicken categorized as non-pork meat flosses are more collected into one cluster, which is shown by the relatively short distance between these two cluster types compared to that in the pork–non-pork cluster case. Increasing the window number leads to the larger distance between two different clusters for all possible meat floss sample correlations.

Figure 4a–d shows that increasing number of windows reduces the cluster size and overlapping, which consequently enlarges the distance between clusters. This phenomenon was found in all possible cluster correlations (i.e., beef–chicken, beef–pork, chicken–pork, and pork–non-pork distances) for different extracted features (maximum, minimum, mean, and median). Thus, clear classification of different clusters can be facilitated enhancing the learning model performance. Here, the separation distance between pork and non-pork cluster centers (see Fig. 4d) tends to be larger than that between two non-pork meat flosses (i.e., beef and chicken in Fig. 4a). This has revealed that beef and chicken categorized as non-pork meat flosses are more collected into one region and separated from the pork cluster. The results of this overlapping in low window number are in accordance with Table 4, where the same volatile compounds (i.e., 1-methylhexyl hydroperoxide and 4-ethyl-octane) were detected from both beef and chicken meat floss samples. From a statistical point of view, this finding is also supported by the PCA and LDA results in Figs. 2 and 3, where the beef and chicken data are more accumulated to each other, and the pork data are more separated from the other two labels (beef and chicken). In other words, a significantly different pattern was detected between non-pork (beef and chicken) and pork meat flosses. This investigation is therefore important to understand and gain insight on the data pattern behavior for a specific time window.

A wide variety of pattern recognition methods can be used to interpret e-nose data. Nevertheless, selecting a suitable method for analyzing a specific type of e-nose data is still challenging^73^. This issue arises due to differences in sensor sensitivity, selectivity, and environmental condition. Additional problems may come from low sensor stability and reproducibility resulting in output sensing variations. Thus, each target class detected with an electronic nose has its unique challenges^74^. In our case, besides LDA, other supervised learning methods (i.e., QDA, k-NN, and RF) were also applied to evaluate the e-nose performance in identifying and classifying the meat flosses. In chemometrics studies, the percentage of correctly classified test sets is used to measure performance of the learning algorithm. This comparison is often used empirically to find the optimal method. However, in reality, the selection of appropriate learning methods should depend on the distribution of samples in variable space^75^.

The accuracy values of all supervised learning models (LDA, QDA, k-NN, and RF) used in the meat floss investigation are listed in Table 3. Here, we compare the results from all four extracted features (maximum, minimum, mean, and median) that were preprocessed with five windows. Internal validation was used to build an accurate model by taking 75% of the overall data (225 samples) and resampling 10-fold cross validation with 10 times repetition. The validation accuracy results range from 93.4% to 99.9%. External validation (testing) was employed to test data testing by taking 25% of the overall data (75 samples). Testing accuracy results range from 89.3 to 100%. We compare all window conditions: without window (W0), with one window (W1), and with two to six windows (W2–W6). We found that the model with five windows (W5) was able to classify samples with the highest accuracy. This is also supported by the results of the LDA plot where the three samples can be completely clustered. Thus, for accuracy value comparison, Table 3 has only listed the performance of learning models with W5. Nevertheless, the complete list of validation analysis results for all the investigated window numbers (W0–10) can be seen in Supplementary Table 1.

**Table 3:** Accuracy results of four different supervised learning models with five windows.

Model	Accuracy (%)							
	Maximum		Minimum		Mean		Median	
	Validation	Testing	Validation	Testing	Validation	Testing	Validation	Testing
LDA	99.9	100	99.7	100	99.6	100	99.4	100
QDA	97.4	93.0	95.9	99.0	98.5	93.0	96.0	92.0
k-NN	94.8	92.0	97.0	93.3	93.4	89.3	93.3	92.0
RF	98.6	97.3	98.7	100	97.5	96.0	97.5	100

The four models were analyzed by different characteristic extraction methods (maximum, minimum, mean, and median). Validation is generated with 75% of the overall data to train the model, while testing is generated to evaluate the model using 25% of the overall data.

There is a deviation in the performance during cross-validation due to the combination of training and validation data that are used for different k-fold iterations. The most stable models were found by monitoring this deviation. In our case, a model with the lowest deviation value was able to maintain its performance for several data variations. Cross-validation with different data combinations showed the behavior of model in the fields. Here, the deviation on the cross-validation was used as an indicator for the model stability level. A model with low deviation value in cross-validation was more consistent in performance than those with high deviation value. The values of mean and deviation metrics during testing were close to their values during cross-validation.

Based on the accuracy results of all models presented in Table 3, it is obvious that different learning models demonstrate their best performances with altered preprocessed features. First, the LDA model performed best in classifying meat floss samples with an extracted feature of maximum value having five windows. Its validation and testing accuracies achieved up to 99.9 and 100%, respectively. Second, the QDA model provides its best accuracy on preprocessing mean value with validation and testing accuracies of 98.5 and 93%, respectively. Third, the k-NN model has a parameter of the number *k* in providing its best classification. Using k = 5, five windows, and extracted feature of minimum value, validation and testing accuracies of 97.0% and 93.3%, respectively, can be obtained by the k-NN model. Fourth, for the RF, mtry is required as a parameter to specify the number of variables that are randomly collected at each split time. The data analysis based on RF model results in the highest validation and testing accuracies of 98.7% and 100%, respectively, when mtry = 6 and extracted feature of minimum in five windows are employed.

Among all tested learning algorithms, the LDA model has been the most superior and suitable method to classify three different types of meat floss samples. It clearly discriminated the beef and the chicken classes from their pork counterpart. Moreover, in the configuration of five windows (W5), the LDA model reached validation and testing accuracies of >99% for all extracted features of maximum, minimum, mean, and median values. This excellent performance was not rivaled by other three learning models (QDA, k-NN, and RF). However, again, selection of the learning algorithms depends on distribution of meat floss samples in variable space. Thus, it is always beneficial to evaluate more than one machine learning model in the data analysis of e-nose, especially during its development phase. Hence, we can achieve the optimum classification results.

### Analysis of chemical compounds

**Fig. 5:**
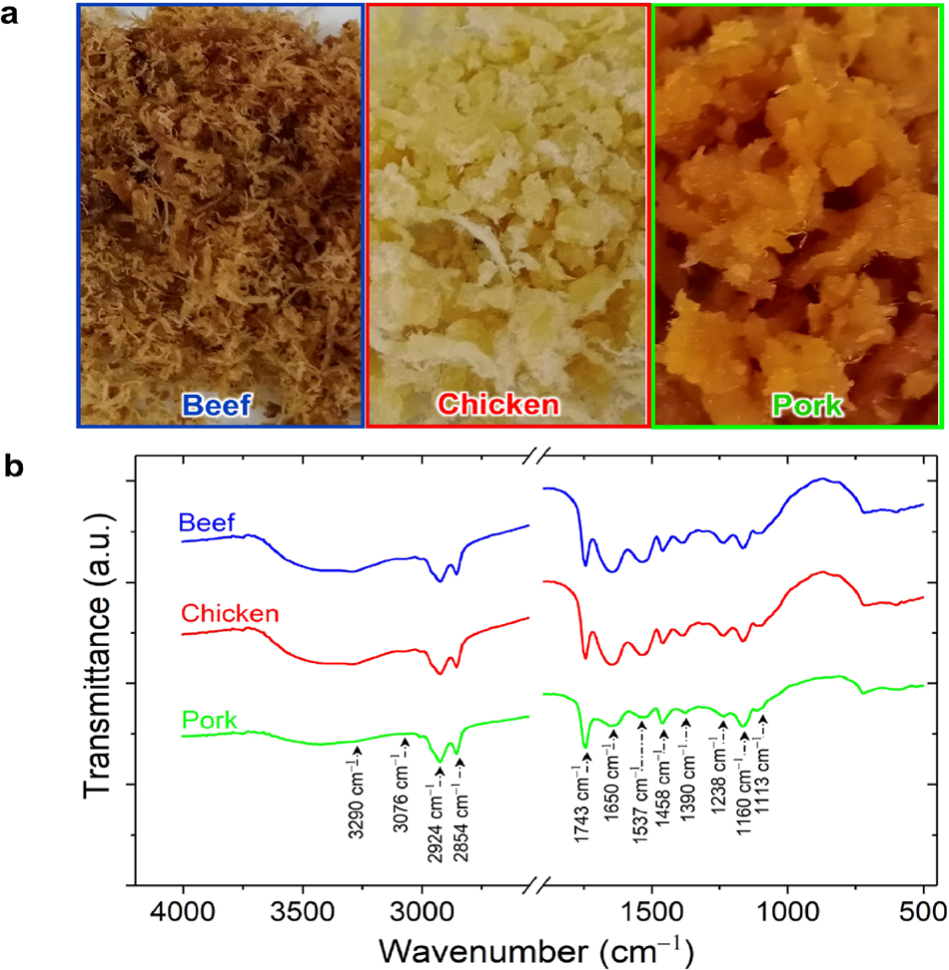
Fourier-transform infrared (FTIR) spectroscopy results of meat flosses. **a** Photographs and **b** FTIR spectra of the meat floss samples (i.e., beef, chicken, and pork meat flosses).

In addition to the analysis using e-nose, products made from beef, chicken, and pork flosses (see Fig. 5a) have also been characterized by Fourier-transform infrared (FTIR) spectroscopy to identify their chemical compositions. Figure 5b shows the FTIR spectra of three different samples of meat flosses (beef, chicken, and pork) at a frequency range recorded between 4000 and 400 cm^−1^. Similar FTIR spectra have been observed in all three samples, showing typical absorption spectra of edible fats and oil. These results are consistent with those obtained in previous study^76^. The strong and wide bands observed at 3290 cm^−1^ are related to N−H and O−H stretching vibrations, while the small bands observed at 3076 cm^−1^ are due to the N−H stretching vibration. These two peaks are related to the protein of the sample. The peak of 2924 cm^−1^ is thought to be the result of the C_sp3_−H stretching of the ethylene chain and the alkyl groups associated with unsaturated lipids. Meanwhile, the vibration of C−H stretching at 2854 cm^−1^ corresponds to the saturated lipids in the meat sample. Another strong peak was also observed at 1743 cm^−1^ due to the C=O carbonyl stretching of ester (triglyceride ester).

The spectra obtained from all three samples also show protein characteristics between 1700 and 1500 cm^−1^, which are similar to the beef, chicken, and pork samples reported in another study^77^. All samples display strong peaks of 1650 and 1537 cm^−1^ that correspond to the amide I band (C−O stretching, C=N starching, and N−H bending of protein) and the amide II band (C−N stretching and N−H bending of peptide bonds). In the FTIR fingerprint area (1450 to 600 cm^−1^), meat floss samples also have the same peaks in 1390, 1238, and 1160 cm^−1^, which are due to fatty acid, phospholipid, and lipid ester, respectively.

Again, all peaks observed in the FTIR spectra of meat floss samples (beef, chicken, and pork) are well consistent with those obtained in earlier studies^77^. These results support PCA-based analysis of the e-nose data (see Fig. 3 and Supplementary Fig. 4). The three different meat flosses (beef, chicken, and pork) remain overlapping due to the same functional groups and similar volatile compounds (see Table 4). The three samples contained volatile compounds: hydrocarbons, ether, and alcohol. In case of hydrocarbons, S1, S2, S4, S5, S7, and S8 could detect 2,4-dimethylhept-1-ene, 4-methyl-1-decene, (E)-4-dodecene, (Z)-5-tridecene, and 3-trifluoroacetoxytridecane. In the meantime, S1, S3, S7, and S8 detected the 6-methylheptyl vinyl ether. Finally, S3 detected various types of alcohols (e.g., 2-butyl-1-octanol, 2,4-di-tert-butylphenol, ethyl iso-allocholate, 6,11-dimethyl-2,6,10-dodecatrien-1-ol). However, an interesting phenomenon is observed with a wavenumber of 1743 cm^−1^, which is related to the fatty acids in the sample. For this particular peak, the peak intensity observed in pork samples is significantly higher than in chicken and beef samples. On the contrary, the peak intensity of the pork sample is lower at 1650 cm^−1^ wavenumber, which further confirms that the protein content of pork floss is lower than those of beef and chicken flosses. The results of the FTIR measurement show that the three different samples (beef, chicken, and pork) contain different fat and oil functional groups, so it is confirmed that the meat products used in the experiment were produced separately during production of the flosses. In other words, they are not mixed in a single composition. Thus, pure beef, chicken, and pork flosses could be obtained. Again, FTIR spectroscopy is an ideal chemical compound analysis technique to complement e-nose. This method can also be used to analyze lipids and distinguish between fat and oil^76^.

**Table 4:** Gas chromatography-mass spectroscopy (GC-MS) results of meat flosses.

Retention time (min)	VOC type	Molecular formula	Volatile	Detected in			Sensors
				Beef	Chicken	Pork	
4.68	2,4-dimethylhept-1-ene	C_9_H_18_	Hydrocarbon	Yes	Yes	Yes	S1, S2, S4, S5, S7, S8
7.83	1-methylhexyl hydroperoxide	C_7_H_16_O_2_	Hydrocarbon	Yes	Yes	No	S1, S2, S4, S5, S7, S8
9.06	4-ethyl-octane	C_10_H_22_	Hydrocarbon	Yes	Yes	No	S1, S2, S4, S5, S7, S8
9.18	4-methyl-1-decene	C_11_H_22_	Hydrocarbon	Yes	Yes	Yes	S1, S2, S4, S5, S7, S8
11.12	(E)-4-dodecene	C_12_H_24_	Hydrocarbon	Yes	Yes	Yes	S1, S2, S4, S5, S7, S8
11.24	6-methylheptyl vinyl ether	C_10_H_20_O	Ether	Yes	Yes	Yes	S1, S3, S7, S8
17.57	2-butyl-1-octanol	C_12_H_26_O	Alcohol	Yes	Yes	Yes	S3
17.80	(Z)-5-tridecene	C_13_H_26_	Hydrocarbon	Yes	Yes	Yes	S1, S2, S4, S5, S7, S8
18.02	3-trifluoroacetoxytridecane	C_15_H_27_F_3_O_2_	Hydrocarbon	Yes	Yes	Yes	S1, S2, S4, S5, S7, S8
18.10	6-methyl-octadecane	C_19_H_40_	Hydrocarbon	No	Yes	No	S1, S2, S4, S5, S7, S8
23.19	2,4-di-tert-butylphenol	C_14_H_22_O	Alcohol	Yes	Yes	Yes	S3
23.53	methoxyacetic 2-tridecyl ester	C_16_H_32_O_3_	Ether	No	Yes	No	S1, S3, S7, S8
29.73	trans-2-dodecen-1-ol	C_12_H_24_O	Alcohol	No	No	Yes	S3
33.77	octadecanal	C_18_H_36_O	Aldehyde	No	No	Yes	S1, S2, S5 S7, S8
38.71	9-octadecenal	C_18_H_34_O	Aldehyde	No	No	Yes	S1, S2, S5 S7, S8
41.79	12-methyl-E,E-2,13-octadecadien-1-ol	C_19_H_36_O	Alcohol	No	No	Yes	S3
45.50	ethyl iso-allocholate	C_27_H_48_O_5_	Alcohol	Yes	Yes	Yes	S3
52.37	6,11-dimethyl-2,6,10-dodecatrien-1-ol	C_14_H_24_O	Alcohol	Yes	Yes	Yes	S3

The similarities and differences in samples of beef, chicken, and pork meat flosses are indicated by time retention in GC-MS. The compounds in the samples are indicated by the presence of peaks (intensity) from the main GC, which are then analyzed by MS. Based on molar mass data and database available in the GC-MS library system, VOC types can be defined.

The chemical compositions of the meat floss samples were further analyzed using gas chromatography coupled with mass spectroscopy (GC-MS), where the results are listed in Table 4. The similarity of the samples was observed by the similar retention time peak. The peak was further analyzed by mass spectroscopy. In general, meat floss samples contain compounds of hydrocarbons and alcohol. Furthermore, some ethers and aldehydes were also found.

Most compounds detected by GC-MS overlapped among the three meat floss samples (beef, chicken, and pork) that were also observed by initial unsupervised assessment using PCA (see Fig. 2). Compounds detected in all meat floss samples include 2,4-dimethylhept-1-ene, 4-methyl-1-decene, (E)-4-dodecene, 6-methylheptyl vinyl ether, 2-butyl-1-octanol, (Z)-5-tridecene, 3-trifluoroacetoxytridecane, ethyl iso-alcoholate, and 6,11-dimethyl-2,6,10-dodecatrien-1-ol. Moreover, some compounds occurred only in beef and chicken samples (i.e., 1-methylhexyl hydroperoxide and 4-ethyl-octane), which can illustrate the overlapping of beef and chicken samples in the supervised classification using LDA (see Fig. 3). Both beef and chicken samples are on the left side of the diagram, while pork sample is on the right side. Fortunately, there are some compounds observed only in chicken samples (e.g., 6-methyl-octadecane and methoxyacetic 2-tridecyl ester). These compounds are believed to be responsible for further discrimination in samples of beef and chicken. In addition, the separation of pork group can be made easier because several compounds are observed only in the pork sample (i.e., trans-2-dodecen-1-ol, octadecanal, 9-octadecenal, and 12-methyl-E,E-2,13-octadecadien-1-ol).

The main volatile compounds detected by the e-nose come mainly from alcohol, ester, and acid hydrolysis products of triglyceride. The GC peak assignment focuses on possible fragments. We may have discovered aldehydes and alkanes. However, their concentration is low. The differences in meat flosses may come from structural isomers, especially alcohol, acids, and alkene. The aromatic components should be rare in the meat. Beef and chicken have similar group of compounds, namely hydrocarbons and alcohol. In accordance with previous research^78^, meat volatile compounds mainly comprise alcohol, aldehydes, ketones, esters, and hydrocarbons. Meanwhile, the main pork products are aldehyde (e.g., dodecanal and 9-octadecanal), which are not present in beef and chicken^79^. From the FTIR data, the C=C bond is more detectable in chicken and beef, which is well consistent with the GC-MS data. According to the above-mentioned results, e-nose is more likely to be an alternative food authentication instrument than GC-MS and FTIR, especially for quickly discriminating pork product from its other meat counterparts (non-pork samples).

## Methods

### Meat floss sample preparation

Three different meat floss materials (i.e., beef, chicken, and pork) were used and processed separately in this study. The materials (meats) were purchased at a traditional market in Sleman, Yogyakarta, Indonesia. All breeds of meats were obtained from the local Indonesian animal species that were ready to be consumed by humans. The meats were taken from local chickens, cows, and pigs that had ages of 45–75 days, 2–4 years, and 4–6 months, respectively. The selected parts were pure meats (only meat cuts without muscles), where neither fat nor bone was added into the composition. For chicken, the pure meats were originally from two body parts (breast and thigh), which were divided into five pieces. For beef and pork, the pure meats were loin cuts, which were divided into four pieces.

The meat cuts were processed to form meat flosses in the laboratory located at the Faculty of Animal Science, Universitas Gadjah Mada (UGM), Yogyakarta. Meat flosses were carefully prepared. Each type of meat (beef, chicken, or pork) with a weight of 500 g was boiled and shredded to form fibers. The shredded meat was fried to dry, in which afterward a spinner was used to remove the water in the obtained meat floss. This process resulted in fried meat floss containing either pure beef, chicken, or pork. Thus, there was no floss product comprising mixed meats in this case.

After their production processes, the meat flosses were prepared as the samples for three different tests (i.e., e-nose, FTIR, and GC-MS). First, for e-nose assessment, we employed a total number of 300 meat floss samples (i.e., 100 samples of beef meat floss, 100 samples of chicken meat floss, and 100 samples of pork meat floss), where each sample has a weight of 2.0 g measured by a TL Series digital scale (a professional digital mini scale with a capacity of 50 g). We characterized 100 samples for each meat floss variant (total weight of 200 g) to provide sufficient data for learning-based classification and to ensure the reliability of the analyzed data. The e-nose measurements of meat floss samples were carried out at room temperature. Second, for FTIR preparation, we used KBr pellets at room temperature using spectrophotometer FTIR Shimadzu Prestige 21. Here, for each meat floss type, we prepared samples with a total weight of 150 g for FTIR measurement. Third, to enable the GC-MS measurement for identifying the volatile compounds in meat flosses, the samples were extracted with methanol. For the GC-MS test, we prepared 150 g of sample for each meat floss type.

### E-nose setup for meet floss investigation

After becoming meat flosses, the samples were tested with the e-nose. The micropump was used to flow the aroma molecules from the sampling container to the sensing chamber as well as to purge the aroma out from the sensing chamber in the cleaning process. The aroma of the sample passing through the sensor array changed the electrical properties of the sensor materials. Then, the electrical signal changes were processed with a data acquisition system (DAQ) that comprises 16 bit analog-to-digital converter (ADC) and microcontroller. Each sensor has different responses displayed on a personal computer. In addition, a temperature and humidity sensor was added inside the sensing chamber to monitor the condition that could affect the sensor array responses^16,51,80^.

### Machine learning models

The measured data were processed with various machine learning methods (i.e., unsupervised and supervised learning models) to classify beef, chicken, and pork meat floss samples with the highest possible validation accuracy. Before they could be processed, the data features had to be first extracted. The technique used in the extraction process was time window slicing or so-called windowing, which was created for the detection line in the sensing signals^81^.

The response sensors were multiplied by the used windows (e.g., five windows as shown in Fig. 1e). Then, the resulting time traces were integrated with respect to time referring to^63^. The number of features in each window can be obtained from the following equations:

4 $$A=[{W}_{1},{W}_{2},\mathrm{..}.,{W}_{i}]$$

5 $${W}_{i}=\mathop{\sum }\limits_{k=1}^{N}R({t}_{k}){K}_{i}({t}_{k})\Delta t$$

6 $${K}_{i}({t}_{k})=\frac{1}{1+{(\tfrac{{t}_{k}-{c}_{i}}{{a}_{i}})}^{2{b}_{i}}}$$

where *A* is the feature vector extracted from one sensor comprising four elements, ${W}_{i}$ is the area surrounded by the response sensor and the windowing function curves, $R({t}_{k})$ is the response value at the time *t*_*k*_, ${K}_{i}({t}_{k})$ is the value of the *i*-th windowing function at the time *t*_*k*_, $k=1,2,3,\mathrm{..}.,N$ denoting the sampling points of each curve, Δ*t* is the sampling interval between two sampling points, and the parameters (${a}_{i},{b}_{i},{c}_{i}$) define the width, shape, and center, respectively, of the different windowing functions ${K}_{i}({t}_{k})$^63^.

The data were cleaned by windowing phase with five windows, and various feature extraction methods (i.e., the maximum^63^, minimum^60^, mean^82^, and median values^60^) were performed in each window. All those four statistical parameters are the basic feature extraction methods obtained from sensor signals that have been extracted in the time domain^60^. The maximum value indicates a change in the sensor maximum reaction rate in response to the scent of the sample. The minimum value is the lowest sensor response value of the entire data. The mean value is a comparison between the total number of sensor responses to the amount of data. The median value is the middle value of the overall sensor response. Maximum, minimum, mean, and median value equations are shown in Table 5^60^.

**Table 5:** Feature extraction formulas for maximum, minimum, mean, and median values.

Feature	Formula
Maximum	$Y=\,\max (Y)$
Minimum	$Y=\,\min (Y)$
Mean	$Y=\tfrac{sum({Y}_{(t)})}{N}$
Median	$Y=\tfrac{{Y}_{\tfrac{N}{2}}+{Y}_{\tfrac{N}{2}+1}}{2}$

Time window slicing was implemented to create different window numbers (W0–W10) in the obtained signals. In case of no window (W0), the overall data were retrieved from 0 to 260 s. Meanwhile, for case of window number other than 0 (W1–W10), the data were retrieved from 20–200 s and subsequently divided into different parts with equal time span for each window.

Feature extraction provides important information from a multidimensional sensor signal collection to obtain optimum results^83^. Basically, the purpose of feature extraction is to take the characteristics of the sample, so that the pattern recognition algorithm can easily recognize one sample from the others. After the features have been extracted from the sensor signals, chemometrics analysis was performed to classify the meat floss samples. Here, the learning investigation started with principal component analysis (PCA), which is an unsupervised pattern recognition method being able to reduce the dimensionality of the data by an orthogonal transformation into principal components (PCs), new variable sets (uncorrelated) that are linear with the original variable sets (possibly correlated variables)^84–86^. Also, PCA is useful for observing the differences and similarities between various samples. In our case, only a few significant components are produced (e.g., PC1 and PC2) and subsequently plotted as a graph^87^.

After performing unsupervised learning model of PCA, the analysis was continued with four supervised learning algorithms (i.e., linear discriminant analysis (LDA), quadratic discriminant analysis (QDA), k-nearest neighbors (k-NN), and random forest (RF)). LDA and QDA, which are boundary detection statistical methods, were used to verify the capability of the e-nose to correctly classify meat floss samples according to their types or origins (beef, chicken, and pork). LDA tries to find a linear combination of features and the best linear fit that can separate two or more sample groups^84^. The LDA maximizes the distance of data between each group and minimize the distance of data in each group. LDA helps locate projection to maximize the separation between samples for class separation^88^. The projection is done by linearly transforming data from a high dimensional space to a low dimensional space, in which finally the decision is made in the low dimensional space^89^. The purpose of the LDA is to change the original dataset to a lower-dimensional space with good sample discrimination. Moreover, the possibility of overfitting is reduced^87^.

The QDA is a simple algorithm and is different from LDA. QDA is used to find quadratic boundaries where a quadratic curve divides the variable space in regions. It computes the variance structures for each class separately, creating a more powerful discrimination rule for classes with different covariance matrices^43^. QDA employs different variance-covariance matrices for each class, which differs it from LDA that only considers a single variance-covariance matrix for all classes^90^.

The k-NN as a non-parametric method is used for classification, regression, and establishment of the classification model classes with different degrees of corruption^73,91^. The main concept of k-NN is to provide a prediction target, calculate the distance or similarity between the predicted target and all samples, select the first *k* sample that is closest to each other, and finally use that sample to choose a decision according to the most votes of *k*^92^. Here, *k* is a positive integer number of neighbors. To calculate the distance between the predicted targets, this model uses the Euclidean distance and selects the dominant class for it^84,93^. Euclidean distance is one of the statistical methods that can be used to calculate the interpoint distances between samples. The usage of the Euclidean distance is shown to be efficient for clustering data^69,70^. Using the closest suitable neighbor search algorithm, k-NN can become computationally traceable even for large datasets^94^.

The RF model is a non-linear statistical ensemble method that constructs and subsequently averages many randomized, decorrelated decisions. RF utilizes the constructed model to estimate the content of samples by exploiting historical data^64^. The method can be used not only for selecting variables (i.e., for understanding the important variables that are used for prediction), but also for solving classification and regression problems with strong abilities to eliminate the noise and to avoid overfitting^64,73^. In RF model, hyperparameter tuning is one of the methods utilized to configure the model parameters and increase the model performance. Nonetheless, a careful attention has to be given when applying this approach because hyperparameter tuning may lead to over-optimistic performance estimation if the used step-by-step methods are not precisely suitable for the model and data condition^95^. RF has several advantages in classification (i.e., the ability to be run on large and high dimensional datasets, good accuracy, stable prediction, and feature selection improvement)^96^. For regression tasks, the RF model has several advantages^97^. First, it offers an uncomplicated inclusion or exclusion of predictors based on data availability and user demands. Second, an inclusion of continuous and categorical predictors is possible. Third, it has a comparatively small number of model parameters that must be specified by the user. Fourth, the risk of overfitting can be minimized. Fifth, the automatic computation of a variable importance score is feasible to assess the contribution of individual predictors to the final model.

For statistical analysis, the data matrix was normalized using scaling and centering techniques. After the normalization step, the initial database was divided into two groups (i.e., training and testing data). Training data were used to develop and create a classification model that allowed the best classification performance for the repeated k-fold cross validation (CV) procedure (10 folds x 10 repetitions, which ensured that at each validation run, 25% of the training data were used for validation accuracy). Meanwhile, the testing data were used for testing accuracy (full prediction) of the classification model that was previously created. The overall modeling development and analysis were carried out using open source R statistical software (version 3.5.1) and utilizing CARET, MASS, and the Kernlab library^50^.

### Chemical compound characterization

FTIR spectroscopy can be used to qualitatively analyze the sample based on its functional groups^98^. In this work, it was used to rationalize the results of the e-nose analysis for distinguishing meat flosses based on their origins that are beef, chicken, and pork^99^. For FTIR analysis, the meat floss was dried separately in the oven for 7 min. The sample was ground into the powder and pelleted with KBr. The FTIR spectra were recorded on Shimadzu 21 Prestige FTIR spectrophotometer in the wavenumber range of 400–4000 cm^−1^.

GC-MS analysis was performed to identify volatile compounds released by the samples^100^. It used a Chromeleon software, a carrier gas of ultra-high pure (UHP) helium, an injector temperature of 260 °C, a mobile phase flow rate of 50 mL/min, a split ratio of 50, a front inlet flow of 1 mL/min, a MS transfer line temperature of 250 °C, an ion source temperature of 200 °C, a purge flow of 3 mL/min, a gas saver flow of 5 mL/min, and a gas saver time of 5 min. For the GC-MS analysis, the meat floss had to be preprocessed first during sample preparation. The meat floss was dried in an oven and then ground into the powder. The sample was extracted using pure methanol (>99%). The analysis was done at room temperature of 23 °C and relative humidity of 55%. The GC-MS data were recorded in an HP-5MS UI column with a length of 30 m and an inner diameter of 0.25 mm. Based on their ion molecular mass (M^+^) and fragmentation pattern, the molar mass of the compound was confirmed with the NIST 14 standard library.

### Reporting summary

Further information on research design is available in the Nature Research Reporting Summary linked to this article.

## Supplementary information

Supplemental Material

Reporting Summary

## Data Availability

The authors declare that all data supporting the findings of this study are available in the paper and supplementary information.
