# Supplementary material for: Rapid analysis of meat floss origin using a supervised machine learning-based electronic nose towards food authentication
Source: NPJ Sci Food. 2023 Jun 16;7:31. doi: 10.1038/s41538-023-00205-2 (PMC10275922; doi:10.1038/s41538-023-00205-2)
Supplement: Supplementary file 1 — Supplemental Material [file 41538_2023_205_MOESM1_ESM.pdf]

Supplementary Information

Rapid analysis of meat floss origin using a supervised machine learning-based electronic nose towards food authentication

Linda Ardita Putri[1,2], Iman Rahman[1,2], Mayumi Puspita[1,2,3], Shidiq Nur Hidayat[1], Agus Budi Dharmawan[1,4], Aditya Rianjanu[5], Sunu Wibirama[6], Roto Roto[7], Kuwat Triyana[2,8,*], Hutomo Suryo Wasisto[1]

[1] PT Nanosense Instrument Indonesia, Umbulharjo, Yogyakarta 55167, Indonesia
[2] Department of Physics, Faculty of Mathematics and Natural Sciences, Universitas Gadjah Mada, Sekip Utara PO Box BLS 21, Yogyakarta 55281, Indonesia
[3] Palm Oil Research Center, Jalan Taman Kencana No 1, Bogor 16128, Indonesia
[4] Faculty of Information Technology, Universitas Tarumanagara, Jl. Letjen S. Parman No. 1, Jakarta 11440, Indonesia
[5] Department of Materials Engineering, Institut Teknologi Sumatera, Terusan Ryacudu, Way Hui, Jati Agung, Lampung 35365, Indonesia
[6] Department of Electrical and Information Engineering, Universitas Gadjah Mada, Jl. Grafika 2, Yogyakarta 55281, Indonesia
[7] Department of Chemistry, Faculty of Mathematics and Natural Sciences, Universitas Gadjah Mada, Sekip Utara PO Box BLS 21, Yogyakarta 55281, Indonesia
[8] Institute of Halal Industry and System (IHIS), Universitas Gadjah Mada, Sekip Utara, Yogyakarta 55281, Indonesia
[*] Corresponding author. E-mail: triyana@ugm.ac.id

Contents

1 Electronic nose system S-2
2 Output signals from gas sensor array S-3
3 Window time slicing method S-4
4 Principal component analysis (PCA) S-5
5 Linear discriminant analysis (LDA) S-7
6 Validation models S-9

1 Electronic nose system

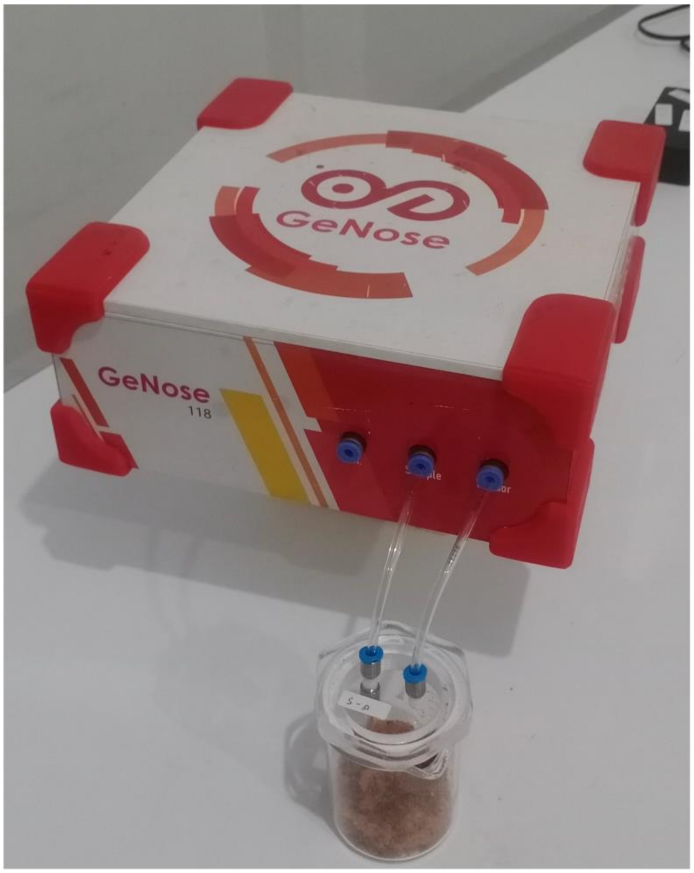

Supplementary Figure 1 Photograph of electronic nose (e-nose) system. E-nose was connected to a sampling container to place the tested meat floss sample.

2 Output signals from gas sensor array

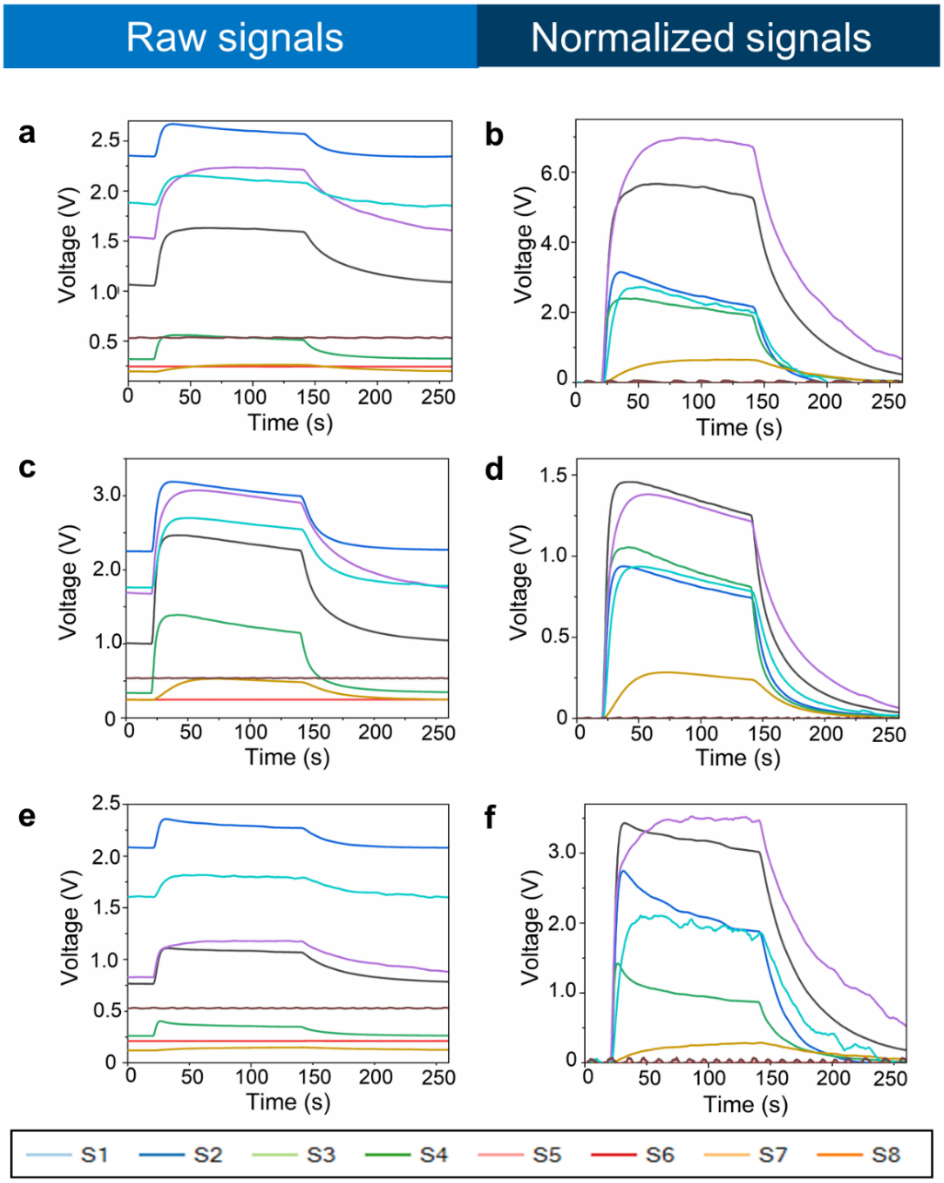

Supplementary Figure 2 Output sensing signals from gas sensor array in the e-nose. The raw and normalized signals generated by eight metal-oxide semiconductor sensors (S1 – S8) while detecting gases released from meat flosses made of **(a,b)** beef, **(c,d)** chicken, and **(e,f)** pork.

3 Window time slicing method

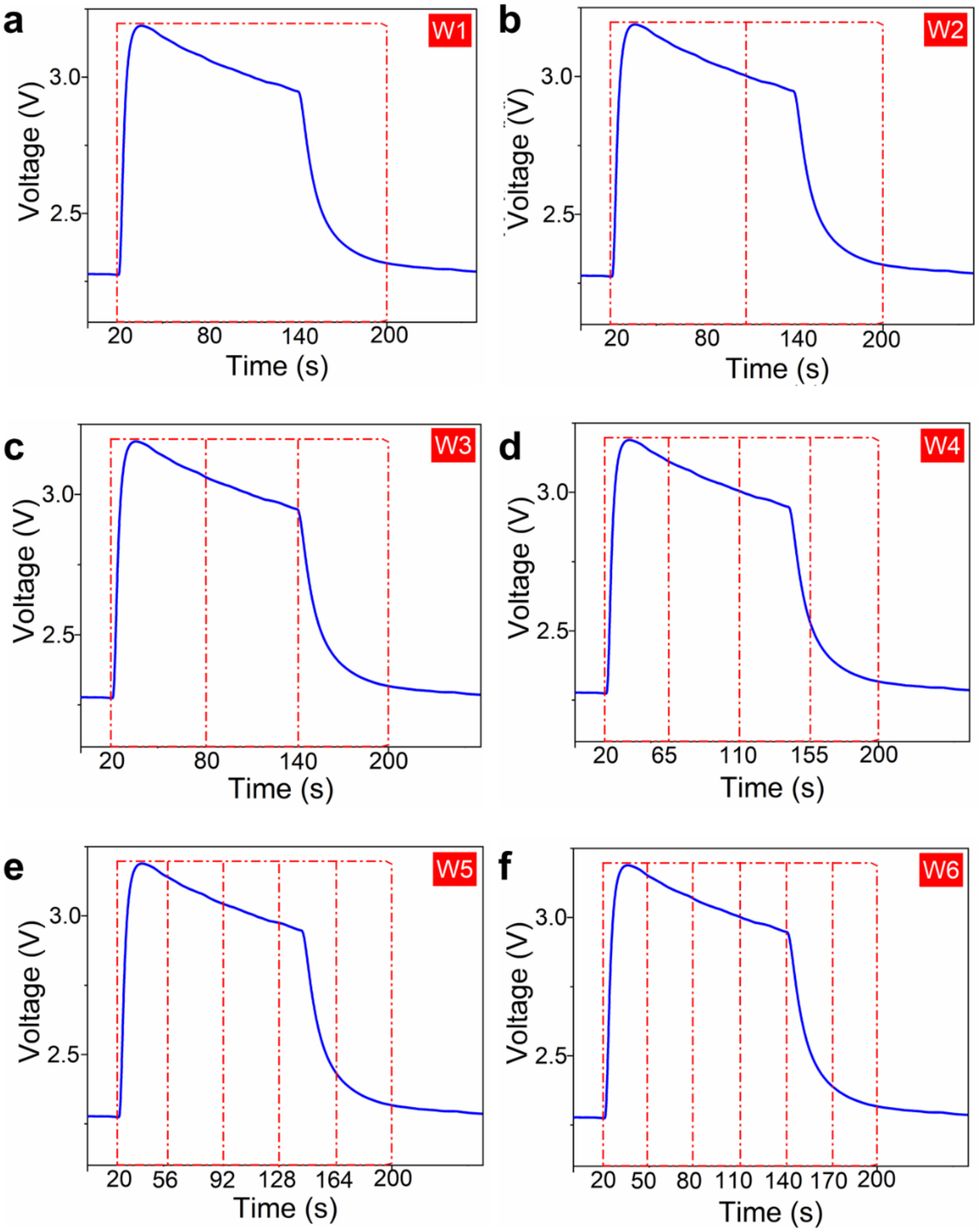

Supplementary Figure 3 Time window slicing method. Different times are applied for slicing the data of senor signals: **(a)** 1, **(b)** 2, **(c)** 3, **(d)** 4, **(e)** 5, and **(f)** 6 windows. For a single or 1 window, the data analysis is carried for time ranging from 20 to 200 s. Meanwhile, in case of more than 1 window (e.g., 2 – 6 windows), a further slicing is performed inside that range with equal span among the sliced data.

4 Principal component analysis (PCA)

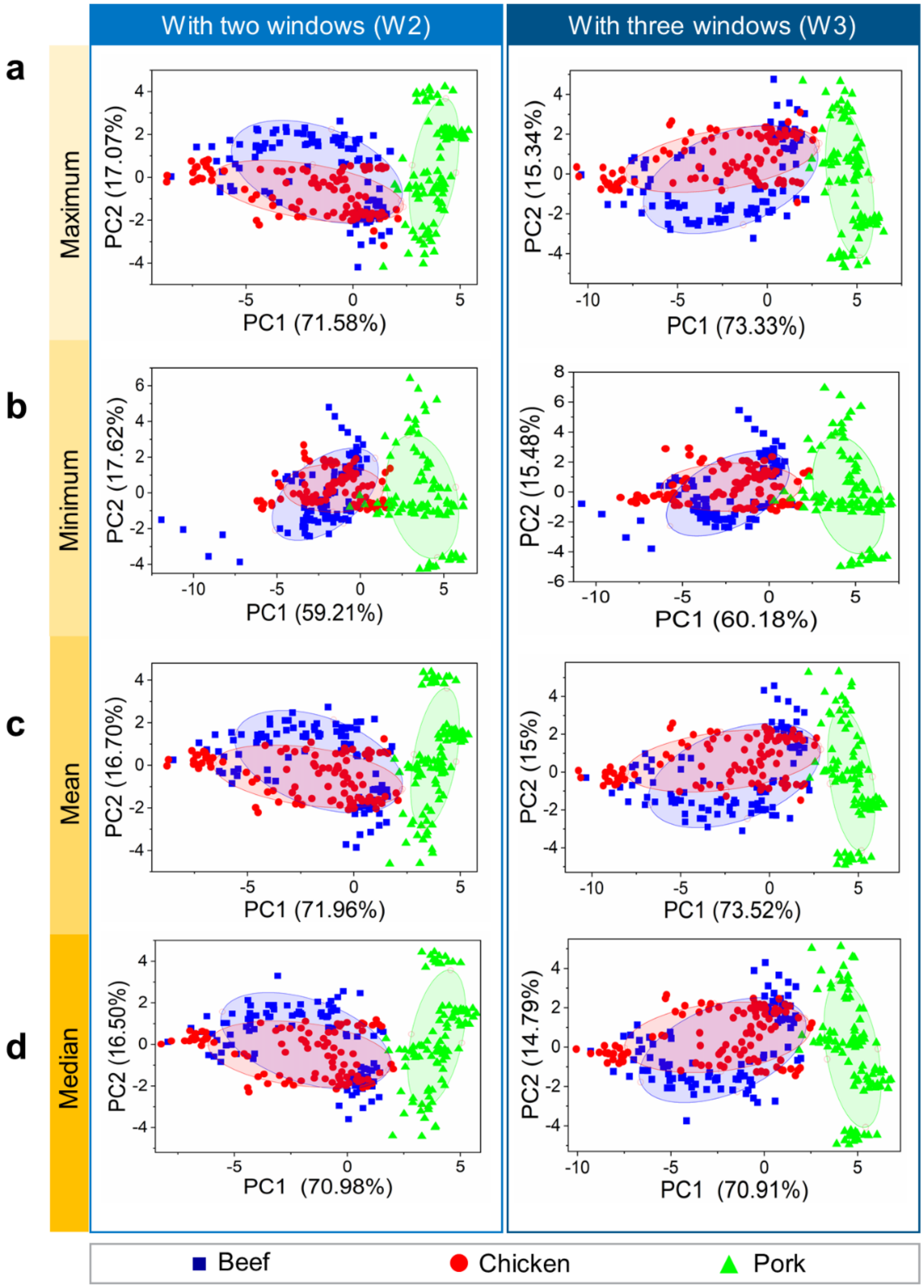

Supplementary Figure 4 Principal component analysis (PCA) with two and three windows. PCA is implemented to analyze output sensing signals that are preprocessed with four different extracted features: **(a)** maximum, **(b)** minimum, **(c)** mean, and **(d)** median values. Time window slicing method is applied to construct different window numbers in the data (i.e., 2 windows (w = 2) and 3 windows (w = 3)).

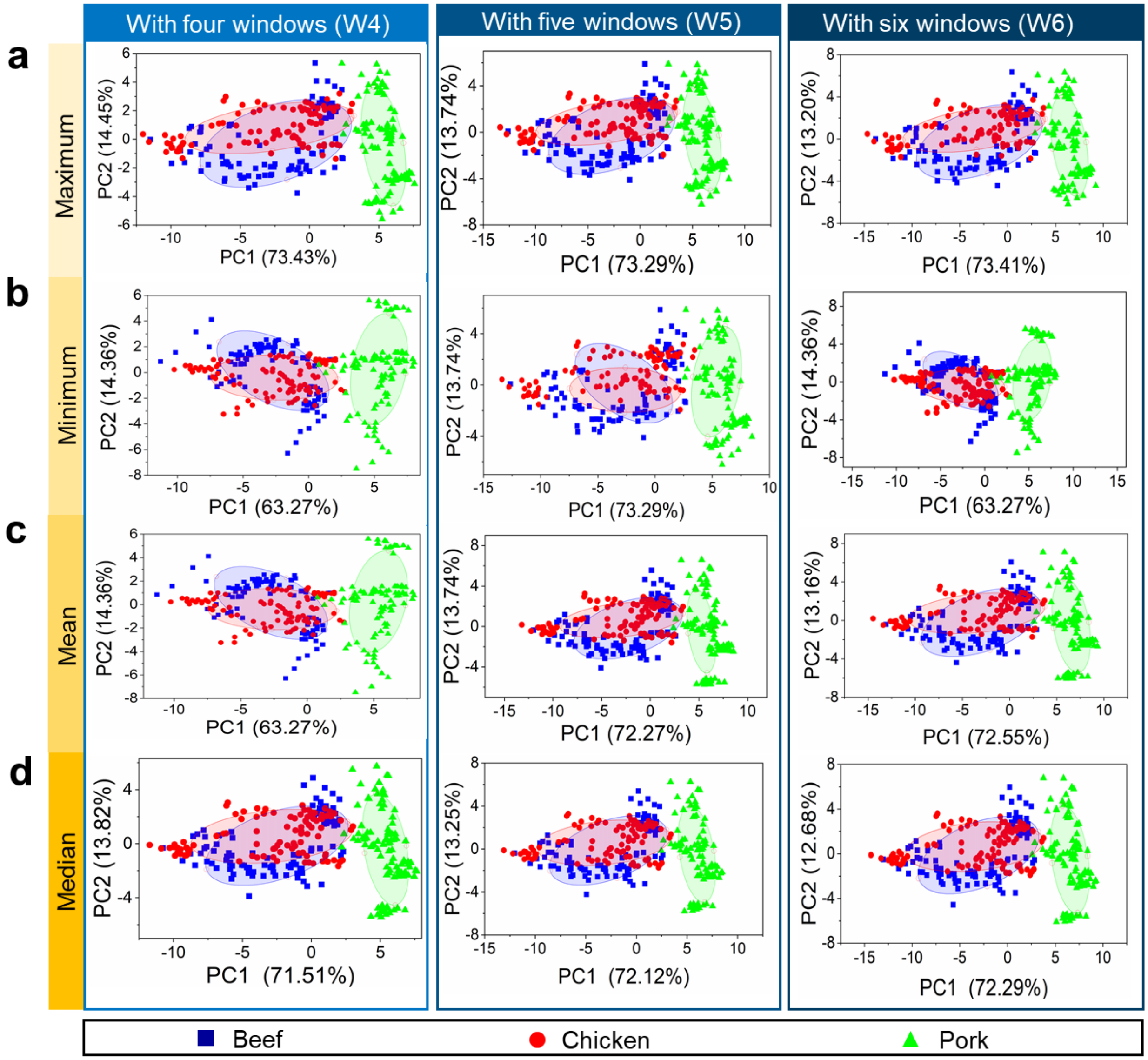

Supplementary Figure 5 Principal component analysis (PCA) with four, five, and six windows. PCA is implemented to analyze output sensing signals that are preprocessed with four different extracted features: **(a)** maximum, **(b)** minimum, **(c)** mean, and **(d)** median values. Time window slicing method is applied to construct different window numbers in the data (i.e., 4 windows (w = 4), 5 windows (w = 5), and 6 windows (w = 6)).

5 Linear discriminant analysis (LDA)

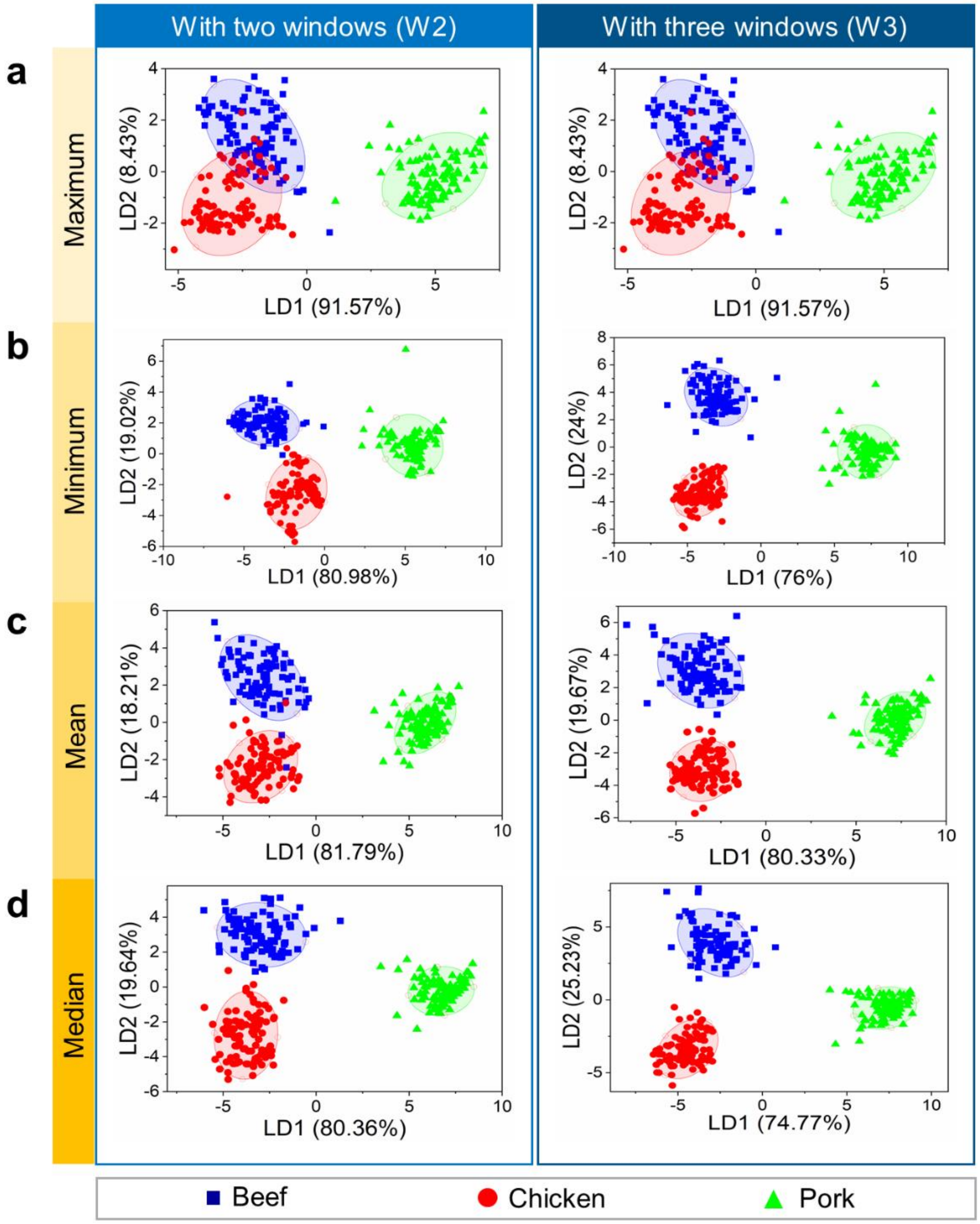

Supplementary Figure 6 Linear discriminant analysis (LDA) integrated with two and three windows. LDA is employed to analyze output sensing signals that are preprocessed with four different extracted features: **(a)** maximum, **(b)** minimum, **(c)** mean, and **(d)** median values. Time window slicing method is applied to construct different window numbers in the data (i.e., 2 windows (w = 2) and 3 windows (w = 3)).

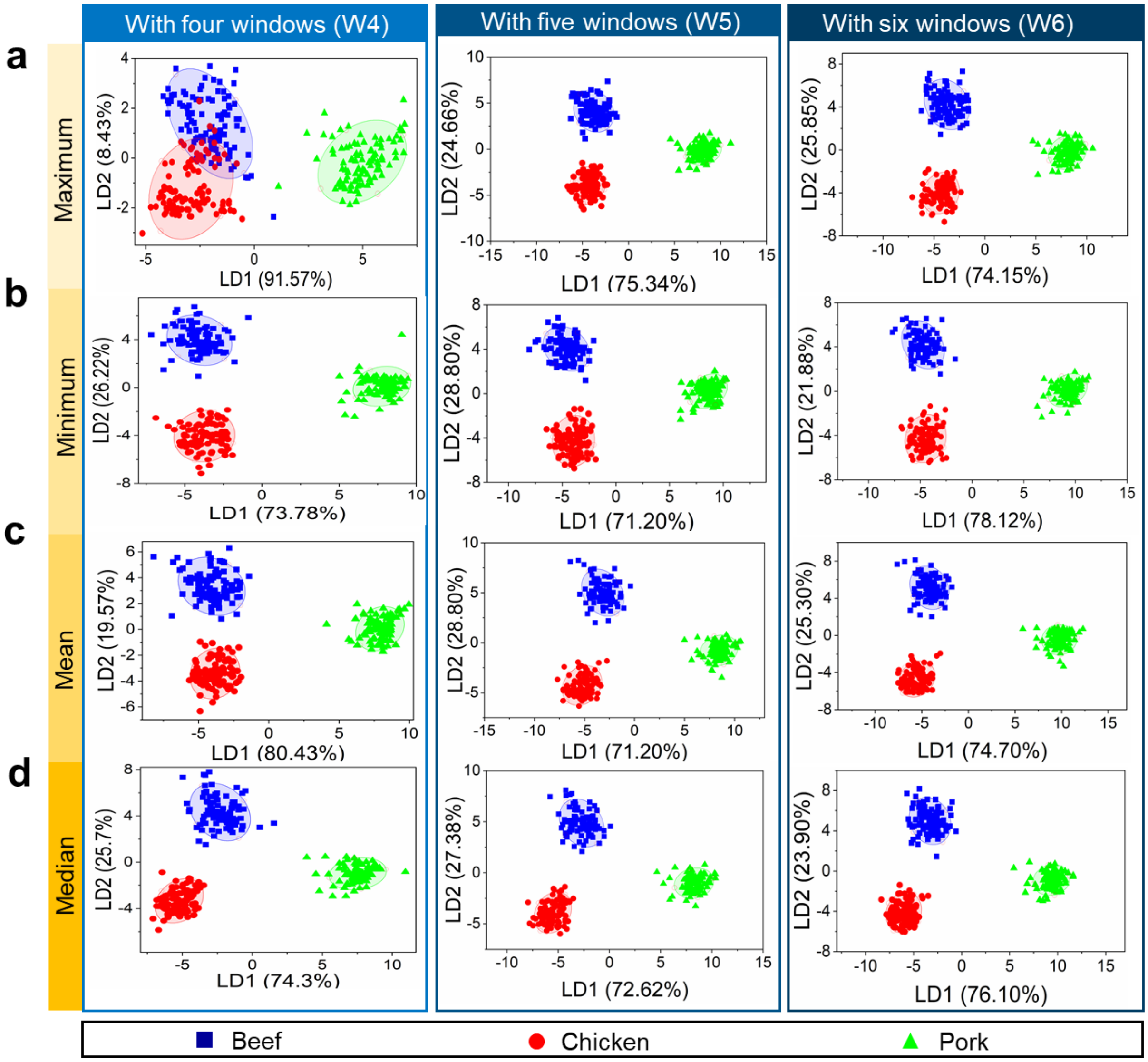

Supplementary Figure 7 Linear discriminant analysis (LDA) integrated with four, five, and six windows. LDA is employed to analyze output sensing signals that are preprocessed with four different extracted features: **(a)** maximum, **(b)** minimum, **(c)** mean, and **(d)** median values. Time window slicing method is applied to construct different window numbers in the data (i.e., 4 windows (w = 4), 5 windows (w = 5), and 6 windows (w = 6)).

6 Validation models

Supplementary Table 1 Accuracy resulting from validation and testing with different supervised learning models (LDA, QDA, k-NN, and RF) and extracted features (maximum, minimum, mean, and median values). Validation analysis is performed for the window number ranging from 0 to 10 windows (W0 – W10). Validation and testing data are used to build and evaluate the learning models, respectively.

Window number	Model	Accuracy (%)							
		Maximum		Minimum		Mean		Median	
		Validation	Testing	Validation	Testing	Validation	Testing	Validation	Testing
0	LDA	95.4	92.0	89.0	90.7	91.5	93.3	93.3	90.7
	QDA	97.3	92.0	96.0	95.0	96.2	97.0	94.8	97.0
	k-NN	93.7	92.0	95.3	90.7	94.0	92.0	94.5	94.7
	RF	96.8	94.7	97.0	94.7	97.0	94.7	100	98.7
1	LDA	95.6	90.7	97.7	90.7	96.9	94.7	97.8	93.3
	QDA	96.4	95.0	98.7	97.0	98.1	97.0	97.5	99.0
	k-NN	93.3	94.7	97.5	96.0	93.2	92.0	93.2	89.3
	RF	96.7	100	99.6	98.7	96.8	97.3	100	97.3
2	LDA	92.3	90.7	99.0	94.7	97.4	97.3	100	97.3
	QDA	97.7	97.0	98.7	97.0	97.6	93.0	97.7	96.0
	k-NN	93.6	92.0	97.0	92.0	93.3	93.3	92.9	94.7
	RF	95.8	98.7	99.8	100	96.9	97.3	100	100
3	LDA	92.3	90.7	99.6	100	98.6	100	99.2	100
	QDA	97.6	93.0	98.8	97.0	97.4	95.0	98.3	95.0
	k-NN	93.2	94.7	97.7	94.7	94.1	93.3	93.8	88.0
	RF	96.5	98.7	99.9	98.7	96.5	97.3	100	93.3
4	LDA	92.3	90.7	99.2	100	98.8	100	100	100
	QDA	97.7	89.0	98.7	100	97.1	96.0	98.0	97.0
	k-NN	94.0	93.3	97.5	94.7	94.0	94.7	93.7	88.0
	RF	97.4	98.7	100	98.7	96.6	97.3	100	100
5	LDA	99.9	100	99.7	100	99.6	100	99.4	100
	QDA	97.4	93.0	95.9	99.0	98.5	93.0	96.0	92.0
	k-NN	94.8	92.0	97.0	93.3	93.4	89.3	93.3	92.0
	RF	98.6	97.3	98.7	100	97.5	96.0	97.5	100
6	LDA	99.4	100	99.2	100	99.3	100	98.7	100

	QDA	97.0	97.0	98.4	100	96.1	99.0	96.8	93.0
	k-NN	94.3	90.7	96.9	93.3	93.6	89.3	94.1	92.0
	RF	98.6	97.3	98.9	100	97.4	98.7	97.4	96.0
7	LDA	99.2	100	99.5	100	99.4	100	98.8	100
	QDA	93.4	93.0	97.2	99.0	81.9	95.0	92.6	92.0
	k-NN	94.7	93.3	96.4	93.3	93.4	88.0	93.7	93.3
	RF	98.8	97.3	99.4	100	97.9	97.3	97.3	97.3
8	LDA	98.4	100	99.9	100	99.2	100	98.6	100
	QDA	64.4	96.0	61.5	92.0	61.8	100	44.2	95.0
	k-NN	94.9	92.0	96.3	93.3	93.6	88.0	93.6	93.3
	RF	98.7	97.3	99.2	100	97.6	96.0	97.1	100
9	LDA	98.5	100	99.3	100	99.0	100	98.3	100
	QDA	66.0	95.0	64.1	99.0	71.8	95.0	70.2	92.0
	k-NN	94.5	92.0	96.1	93.3	93.6	88.0	94.1	92.0
	RF	96.8	100	99.6	98.7	98.2	98.7	98.2	97.3
10	LDA	99.1	100	99.2	100	99.5	100	97.7	100
	QDA	65.0	96.0	69.1	99.0	68.3	95.0	69.5	95.0
	k-NN	94.3	89.3	95.9	93.3	94.0	88.0	93.8	92.0
	RF	97.0	100	99.6	98.7	98.3	97.3	98.2	98.7
